# Expanding the Design Space of Polymer–Metal Organic Framework (MOF) Gels by Understanding Polymer–MOF Interactions

**DOI:** 10.1021/acs.chemmater.4c00112

**Published:** 2024-07-25

**Authors:** Prince Verma, Mark S. Bannon, Mara K. Kuenen, Sanoj Raj, Ankit Dhakal, Kevin Stone, Asa W. Nichols, Charles W. Machan, Yamil J. Colón, Rachel A. Letteri, Gaurav Giri

**Affiliations:** † Department of Chemical Engineering, University of Virginia, Charlottesville, Virginia 22903, United States; ‡ Department of Chemical and Biomolecular Engineering, University of Notre Dame, Notre Dame ,Indiana46556, United States; § Stanford Synchrotron Radiation Lightsource, SLAC National Accelerator Laboratory, Menlo Park, California 94025, United States; ∥ Department of Chemistry, University of Virginia, Charlottesville, Virginia 22904, United States

## Abstract

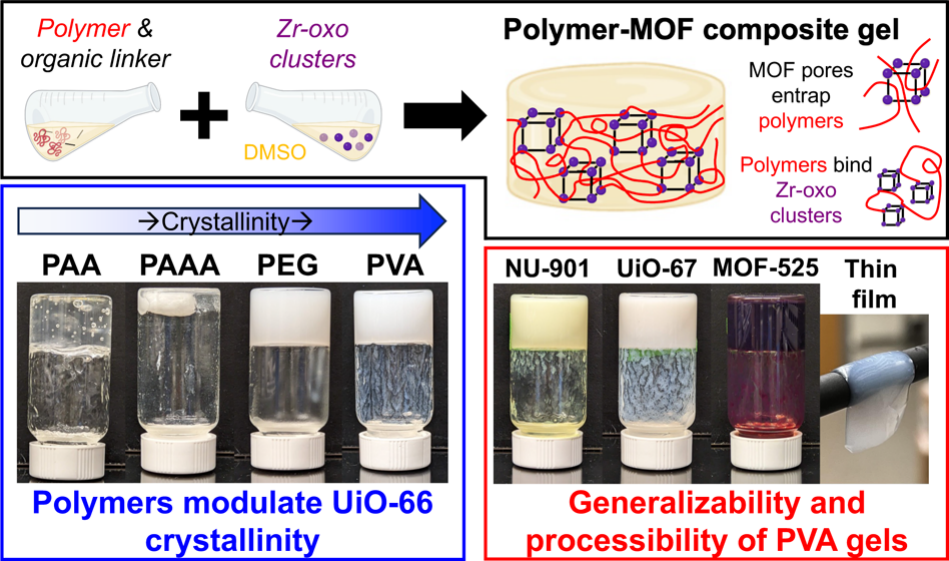

The fabrication of polymer-MOF composite gels holds great potential to provide emergent properties for drug delivery, environmental remediation, and catalysis. To leverage the full potential of these composites, we investigated how the presence and chemistry of polymers impact MOF formation within the composites and, in turn, how MOFs impact polymer gelation. We show that polymers with a high density of strongly metal-binding carboxylic acids inhibit MOF formation; however, reducing the density of carboxylic acids or substituting them with weaker metal-binding hydroxyl groups permits both MOF formation and gelation within composites. Preparing composites with poly(ethylene glycol) (PEG), which does not bind MOF zirconium (Zr)-oxo clusters, and observing gelation suggests that MOFs can entrap polymer chains to create cross-links in addition to cross-linking them through polymer-Zr-oxo interactions. Both simulations and experiments show composite hydrogels formed with poly(vinyl alcohol) (PVA) to be more stable than those made with PEG, which can reptate through MOF pores upon heating. We demonstrate the generalizability of this composite formation process across different Zr-based MOFs (UiO-66, NU-901, UiO-67, and MOF-525) and by spin-coating gels into conformable films. PVA-UiO-66 composite hydrogels demonstrated high sorption and sustained release of methylene blue relative to the polymer alone (3× loading, 28× slower release), and PVA-MOF-525 composite hydrogels capably sorb the therapeutic peptide Angiotensin 1–7. By understanding the influence of polymer-MOF interactions on the structure and properties of composite gels, this work informs and expands the design space of this emerging class of materials.

## Introduction

Metal–organic frameworks (MOFs), crystalline coordination networks containing organic linkers bridged by metal ions/clusters,^1^ can be integrated with polymers to create polymer-MOF composite gels that enable advanced capabilities in optoelectronics,^2^ wound healing,^3−6^ and separations.^7−13^ The functionality and versatility of these composite materials arise from enhanced sorptive capacity,^14^ processability,^15^ and mechanical stability^16,17^ relative to the individual polymer and MOF constituents. For example, synthesizing MOF-808 within metal-cross-linked alginate gels increased methylene blue sorption by an order of magnitude compared to the alginate gels alone.^12^ In another example, forming HKUST-1 MOFs within bentonite clay-cross-linked poly(vinyl alcohol) networks furnished 3D-printable MOF-laden inks.^18^ Additionally, the synthesis of ZIF-8 within a gelatin matrix enhanced the storage modulus of the composite gels (5×) compared to the gelatin alone.^19^ While interactions between MOFs and polymers that facilitate composite gel formation are quite beneficial in these examples, polymer-MOF interactions can also detract from the intended properties of composite gels.

MOF formation requires the coordination of organic linkers to metal ions/clusters, while gel formation in these systems generally relies on cross-linking of polymer chains by the same metal ions/clusters. Therefore, this competition between the organic linker and polymer for binding the metal ions/clusters can disrupt MOF crystallization in composite gels. For example, synthesizing MOF-808 within Zr^4+^-cross-linked alginate gels yielded composite gels with lower crystallinity than MOF-808 particles formed in the absence of alginate.^12^ It is in turn possible that the linkers outcompete polymers for binding the metal ions/clusters, reducing cross-link density in the composite gels.^13^ To gain control over polymer-MOF composite gel properties, we must further understand the competition between linkers and polymers for metal ions/clusters.

To date, many reported polymer-MOF composite gels rely on carboxylic acid (−COOH) containing polymers, such as alginate.^9,11,13,20−22^ However, since many MOF linkers also contain carboxylic acids, competition will likely occur between the carboxylic acids on the polymer and the linker for binding metal clusters. Using polymers with more weakly metal binding functional groups (e.g., amines (−NH_2_) or hydroxyl (−OH)) than carboxylic acid groups can permit crystalline MOF formation without preventing gelation. For example, four crystalline MOFs were formed within chitosan composite gels, suggesting that the chitosan hydroxyl and amine groups did not prevent MOF formation.^8^ Encouragingly, this suggests that polymer molecular details can tune the MOF formation properties and the polymer-MOF interactions and, by extension, the properties of the resulting composites. On the other hand, the MIL-100 MOF did not form within the same chitosan networks,^8^ highlighting that, in addition to the polymer functional groups, it is important to consider the effects of MOF chemistry on MOF crystallinity and formation within composite gels. Studies so far have mainly highlighted the benefits of polymer-MOF combinations. However, comparisons of composite structure and properties (e.g., MOF crystallinity, gel properties, sorption characteristics) with control materials lacking either polymer (i.e., MOF alone) or linker (i.e., metal-cross-linked polymer gels) are needed for understanding the role of molecular details on the structure and properties of these composite gels.

Here, we investigate how the molecular details of polymers and MOFs affect the formation, gelation behavior, crystallinity, and sorption characteristics of their composite gels. Specifically, for these studies, we form composite gels using polymers with different types and densities of functional groups (i.e., carboxylic acids, hydroxyl groups, or neither) and a range of Zr-based MOFs. We then compare the ability of the composite gels, Zr-cross-linked polymer gels (control without MOF linker), and MOF (control without polymer) to sorb and release the small molecule dye methylene blue and the therapeutic peptide Angiotensin 1–7. By understanding the role of molecular details of polymers and MOFs on the properties of composite gels prepared by forming MOFs in the presence of polymers, we expand the design space of polymer-MOF composite gels.

## Results and Discussion

We first studied the impact of polymer chemistry on MOF formation, and the impact of MOF formation on polymer cross-linking. To probe the role of polymer chemistry in MOF formation, we formed MOFs in the presence and absence of poly(acrylic acid) (PAA), poly(acrylamide-*co*-acrylic acid) (PAAA), poly(vinyl alcohol) (PVA), and poly(ethylene glycol) (PEG), and assessed the crystallinity of the resulting MOFs. We note that aside from PEG, these polymers are all simple hydrocarbon chains with functional groups pendant to every other carbon, allowing us to isolate the effects of these functional groups. In turn, to investigate the extent to which MOF formation plays a role in polymer cross-linking, we compared the gelation behavior of Zr-cross-linked polymers, which were formed with in the absence of the organic MOF linker, to that of the polymer-MOF composites.

**Figure 1:**
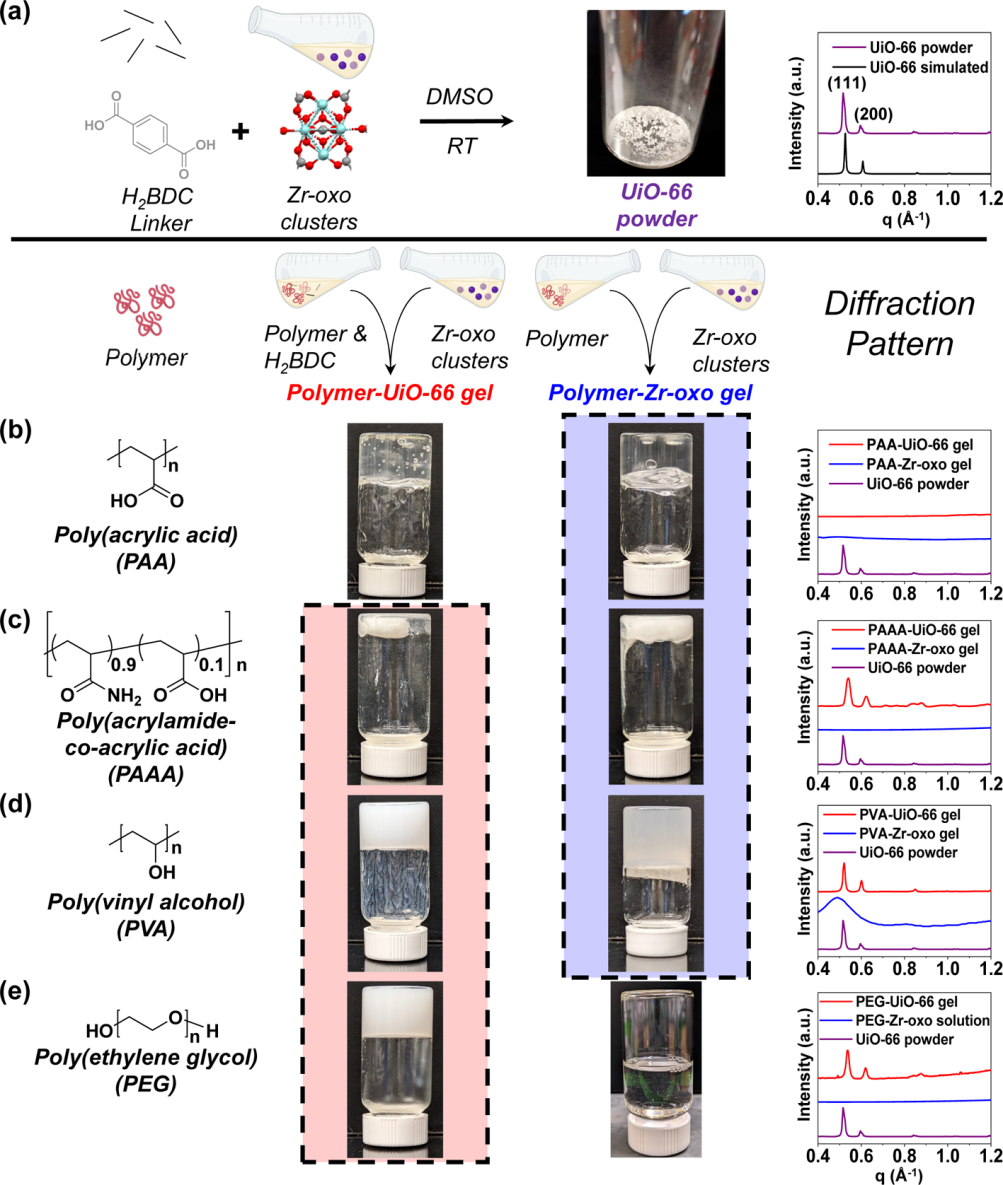
Polymer gelation and MOF formation within polymer-MOF composite gels, varying in polymer functional group chemistry and density. (a) Formation of UiO-66, with the corresponding grazing incidence X-ray diffraction (GIXD) pattern (purple) compared with the simulated pattern (black). In the structure of the Zr-oxo clusters, generated using Mercury,^28^ Zr, O, and C atoms are colored cyan, red, and gray, respectively. Gelation behavior (center) and GIXD patterns (right) of polymer-UiO-66 composite gels (red) and polymer-Zr-oxo gels (blue) synthesized with (b) poly(acrylic acid) (PAA), (c) poly(acrylamide-*co*-acrylic acid) (PAAA), (d) poly(vinyl alcohol) (PVA), and (e) poly(ethylene glycol) (PEG). For PAA, we form gel, but not UiO-66. For PAAA and PVA, we form composite gels containing UiO-66, but those with carboxylic acid-containing PAAA show lower UiO-66 crystallinity, while hydroxyl-containing PVA maintains crystallinity. For PEG, we form composite gels containing UiO-66, but PEG-Zr-oxo mixtures lacking linker do not gel, indicating MOF formation is necessary for gelation. All samples were synthesized in dimethyl sulfoxide (DMSO). Image of the Zr-oxo cluster is adapted with permission from ref (27). Copyright 2022 American Chemical Society.

For evaluating the role of polymer chemistry, we selected the prototypical MOF UiO-66, featuring zirconium (Zr)-oxo clusters linked with benzene dicarboxylic acid (H_2_BDC) linker. Previously finding that preforming Zr-oxo clusters before adding linker accelerates MOF formation,^23,24^ we first synthesized the clusters by dissolving zirconium propoxide (70 wt % in 1-propanol) and acetic acid modulator in DMSO followed by heating at 130 °C for 2 h. Then, we added the H_2_BDC linker as a powder to the Zr-oxo cluster solution, which is transparent (Figure S1a), and stirred at room temperature (RT) for 24 h to form UiO-66 (Figure 1a). As is typical with UiO-66 formation,^25^ the solution turned white within 4–6 h (Figure S1b). We isolated the UiO-66 powder by dialyzing the suspension against DI water, followed by drying the particles at RT in ambient conditions. Scanning electron microscopy (SEM) images of the MOF powder revealed spherical particles ∼30 to 80 nm in diameter, typical of this accelerated MOF formation protocol,^24^ (Figure S2). Grazing incidence X-ray diffraction (GIXD) from the powder shows characteristic diffraction peaks corresponding to scattering from the (111) and (200) planes of UiO-66 (Figure 1a). From the GIXD pattern, we used the Scherrer equation^26^ to calculate the coherence length of UiO-66, reflective of the length scale over which crystalline order persists, as 40 nm.

To form composite gels, we added a MOF linker to the polymer solution before mixing with Zr-oxo clusters. We chose to premix the polymer and linker to promote simultaneous formation of MOF and gel, since premixing linker and Zr-oxo clusters prior to adding polymer might preferentially promote MOF formation and premixing polymer and Zr-oxo clusters prior to adding linker might preferentially promote gelation. We first attempted to form composite gels with a 1:1 molar ratio of linker:metal similar to the MOF alone, but obtained no gelation. Increasing the linker:metal molar ratio to 2:1 yielded gels (Figure S3). A possible explanation is that doubling the linker concentration accelerates UiO-66 crystallization, generating defects (i.e., open metal sites) and availing open Zr-oxo cluster sites for polymers to bind and gel.^27^ Thus, all polymer-UiO-66 composites gels were formed at a 2:1 linker:metal molar ratio. We have included a discussion in the Supporting Information in Section S1b on why using a lower linker:metal ratio was not plausible.

### MOF and Gel Formation in Carboxylic Acid-Containing Polymer Solutions

Anticipating that polymers with carboxylic acid groups would compete most strongly with the carboxylic acid-containing linkers for binding the Zr-oxo clusters, we first attempted to form PAA-UiO-66 composite gels. Adding a solution of Zr-oxo clusters to a solution containing H_2_BDC linker and 2 wt % PAA (*M*_w_ ∼1,033,000 g/mol) in DMSO yielded a self-supporting gel after 24 h at room temperature (Figure 1b, center left). Yet, the gel was transparent, and since UiO-66 formation should produce particles that give the gel a white color, we suspected limited-to-no UiO-66 formation within the gel. Corroborating our visual assessment, GIXD patterns contained no peaks characteristic of the (111) or (200) planes of UiO-66 crystals (Figure 1b, right). Furthermore, unlike UiO-66 alone, which forms spherical particles (Figure S2), we observed no spherical particles in SEM images of the gels (Figure S4). Suspecting that gelation was due to Zr-oxo clusters cross-linking PAA chains, we added a solution of Zr-oxo clusters to a PAA solution containing no linker, which also produced a self-supporting PAA-Zr-oxo gel (Figure 1b, center right). Since UiO-66 did not form in the presence of PAA, presumably due to PAA outcompeting the H_2_BDC linker for binding the Zr-oxo clusters, we next sought to form composites by adding preformed UiO-66 to PAA. While we did observe a small amount of gelation of these physical mixtures of MOF and polymer, we did not observe the homogeneous gelation necessary to pass an inversion (Figure S5a), a result we ascribe to the lower accessibility of Zr-oxo clusters needed for polymer cross-linking.

As PAA, with carboxylic acid groups pendent to every other carbon, inhibited UiO-66 formation, we reasoned that lowering the density of carboxylic acids on the polymer would permit MOF formation simultaneously with gelation. We next attempted composite gel formation with PAAA, a copolymer containing 10% acrylic acid groups randomly dispersed among acrylamide units with *M*_w_ ∼210,000 g/mol (Figure 1c, left). Despite the lower density of carboxylic acids and the lower molecular weight of this polymer, mixtures of PAAA and Zr-oxo still formed gels in the absence of linker (Figure 1c, center right). In the presence of linker, we also observed the formation of a self-supporting gel (Figure 1c, center left). Yet in this case, the opaque white color of this gel was an encouraging indication of UiO-66 particle formation within the gel, which GIXD patterns (Figure 1c, right) and SEM confirmed (Figure S6). While the coherence length of UiO-66 synthesized in the presence of PAAA (26 nm) was slightly lower than that of UiO-66 alone (40 nm), it is apparent that lowering the carboxylic acid density within the polymer permitted simultaneous MOF formation and gelation, suggesting functional group density can modulate MOF crystallinity.

### MOF and Gel Formation in Hydroxyl-Containing Polymer Solutions

Relative to carboxylic acids, hydroxyl groups interact less strongly with Zr-oxo clusters;^29^ therefore, we expected that forming UiO-66 in the presence of poly(vinyl alcohol) (PVA, *M*_w_ ∼146,000–186,000 g/mol, Figure 1d, left) would also furnish composite gels despite the anticipated lower binding affinity of PVA to Zr-oxo clusters. To this end, adding a Zr-oxo cluster solution to a PVA solution in the absence of H_2_BDC linker yielded a self-supporting PVA-Zr-oxo gel (Figure 1d, center right). Upon adding Zr-oxo solution to a mixture of PVA and H_2_BDC linker, the resulting gel exhibited the opaque, white color characteristic of UiO-66 formation (Figure 1d, center left). SEM (Figure S7) and GIXD (Figure 1d, right) confirmed UiO-66 formation, showing UiO-66 particles within the composite, that had a similar coherence length (44 nm) to UiO-66 formed in the absence of polymer (40 nm).

Given that PVA yielded more crystalline UiO-66 within composite gels compared to both carboxylic acid-containing polymers, we next sought to determine the effects of PVA molecular weight and concentration on composite gelation behavior. Varying the concentration of PVA (*M*_w_ ∼146,000–186,000 g/mol) used to form PVA-UiO-66 composites from 0.1 to 3.0 wt %, we obtained self-supporting gels at and above 2.0 wt % (Figure S8). Reducing PVA molecular weight to *M*_w_ ∼31,000–50,000 g/mol required a higher polymer concentration (3 wt %) for gelation (Figure S9). Further reducing PVA molecular weight to *M*_w_ ∼9,000–10,000 g/mol did not result in gelation even at 4 wt % PVA (Figure S10). Together, these results suggest that polymer chains must be long enough or at a high enough concentration in solution to bridge multiple MOF particles (or Zr-oxo clusters) for gelation to occur (Figure S11).

### Poly(ethylene glycol)-MOF Composites

Since hydroxyl-containing PVA facilitated the formation of more crystalline UiO-66 compared to carboxylic acid-containing polymers, we next further reduced the interactions between polymer and Zr-oxo clusters by using poly(ethylene glycol) (PEG, *M*_v_ ∼100 000 g/mol), which only contains hydroxyls at the chain ends. Unlike the polymers with carboxylic acid and hydroxyl pendant groups, mixing PEG with Zr-oxo clusters did not produce gels (Figure 1e, right center). However, adding a solution of Zr-oxo clusters to a mixture of PEG and H_2_BDC linker in DMSO did yield a self-supporting gel with the opaque, white color characteristic of MOF formation (Figure 1e, left center). SEM (Figure S12) and GIXD (Figure 1e) revealed the presence of UiO-66 particles with a comparable, yet slightly lower coherence length (32 nm) to UiO-66 synthesized in the absence of polymer (40 nm). Since Zr-oxo does not cross-link PEG, the observation of gelation during MOF formation suggested the exciting possibility that MOFs may cross-link polymers by physically entrapping them as they form. To further probe the possibility that PEG is simply entrapped in the composites, we heated the PVA- and PEG-based composites to 40 °C for 24 h. After cooling back down to room temperature, the PEG-UiO-66 composite gel flowed (Figure S13a), while the PVA-UiO-66 composite gel remained intact (Figure S13b).

**Figure 2:**
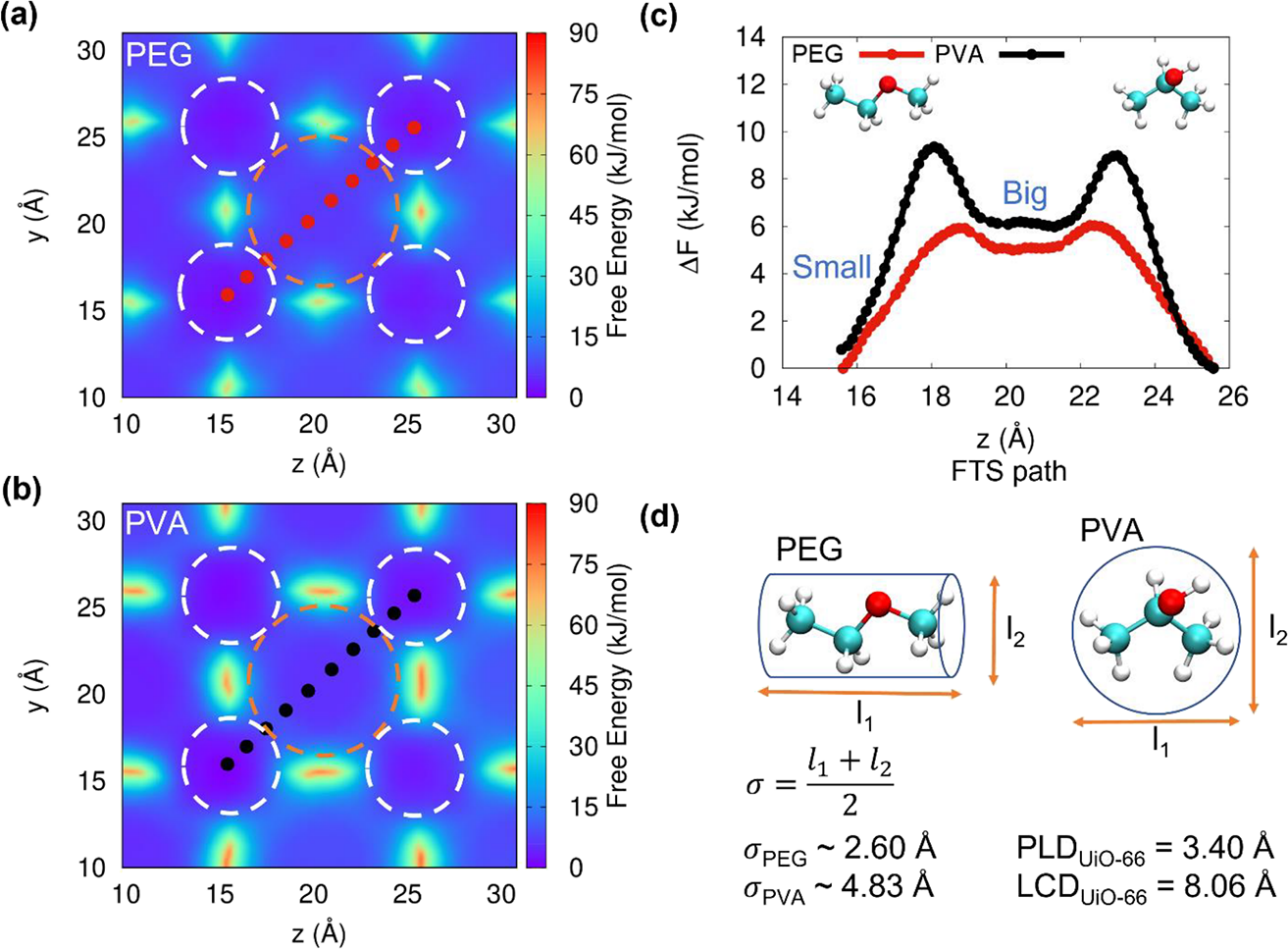
Calculating the energy barriers for monomer mimics of PVA and PEG to traverse UiO-66 pores. For a PEG monomer mimic (a) and a PVA monomer mimic (b), the heat maps show the 2D free energy landscape, i.e., the free energy calculated for the PVA and PEG monomer mimics as a function of position within UiO-66, where the white and orange circles outline the small and large pores, respectively. The dots overlaid on the free energy landscape represent the most probable pathway the monomers traverse between the pores, as determined by the Finite Temperature String (FTS) method. (c) Free energy of PVA and PEG monomer mimics as a function of the *z* coordinate of the FTS-determined pathway. (d) Estimation of the effective diameters (σ) for PEG and PVA monomer mimics and the pore limiting diameter (PLD) of UiO-66. Largest cavity diameter (LCD) represents the size of the large pore as shown by the orange circles.

With experimental evidence showing PVA-based composites to be more thermally stable than PEG-based composites, presumably due to the stronger interactions between PVA and Zr-oxo clusters, we next calculated the relative energy barriers required for PVA and PEG to traverse through the MOF pores. A higher energy barrier would suggest that more energy is required for the polymer to reptate through the MOF pores. For small molecule (1-unit) mimics of PEG (CH_3_OCH_2_CH_3_) and PVA (CH_3_CH(OH)CH_3_), we first calculated the free energy as a function of position of the monomer mimics in UiO-66. These 2D free energy landscapes are shown as heatmaps in Figure 2a,b. Next, we used the finite temperature string (FTS) method to determine the most probable pathway for each monomer mimic to traverse the UiO-66 pores, specifically to travel from a small pore (outlined in white dashed circles) to a larger pore (outlined in orange dashed circles) and back to a small pore. These are shown as dots overlaid with the heat maps in Figure 2a,b. From the 2D free energy landscape, we then obtained the free energy barrier required for the small molecule mimics to traverse those pathways (Figure 2c). We found the free energy barrier for a PVA monomer mimic to travel between the large and small pores of UiO-66 to be ∼9 kJ/mol, whereas that of the PEG monomer mimic was just ∼6 kJ/mol. The lower energy barrier of PEG to traverse the UiO-66 pores is consistent with the experimental finding that the PEG gels flow upon heating and suggests that heating the PEG-UiO-66 composite gels might provide enough energy for PEG chains to traverse through and reptate out of the UiO-66 pores. Conversely, the energy barrier for PVA chains to traverse through UiO-66 pores may be too high to overcome at 40 °C, and thus the gel remains intact. While the stronger interactions between PVA and Zr-oxo compared to PEG and Zr-oxo contribute to the greater stability of the PVA-based composites, simulations account for both chemical affinity and steric interactions. As shown in Figure 2d, the effective diameter of the PEG and PVA monomer mimics are ∼2.60 Å and ∼4.83 Å, respectively, while the pore limiting diameter (PLD) and largest cavity diameter (LCD) of UiO-66 are 3.40 Å and 8.06 Å, respectively. Therefore, PVA may be sterically hindered from traversing the UiO-66 pores, which may also contribute to the higher stability of these composites at 40 °C. This consideration highlights polymer steric bulk as an important future consideration for designing polymers for polymer-MOF composite gels. Together, these experiments and simulations suggest that crystallizing MOFs in the presence of polymers may lead to polymer entrapment. In addition to chemical interactions between the polymer and MOF components, such entrapment of the polymer by the MOF may physically cross-link the polymer, furnishing gel composites.

### Extending PVA-MOF Composite Gel Synthesis to Other Zr-Based MOFs and Processability of the Composite Gel

**Figure 3:**
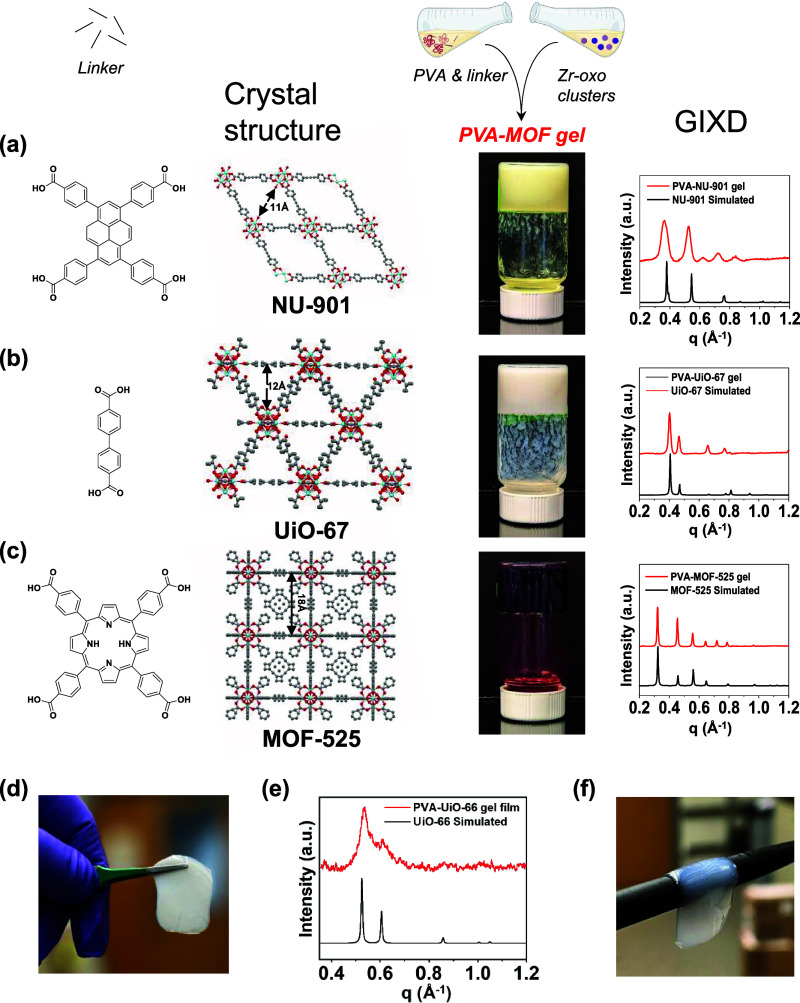
Extension of the composite gel synthesis process to different Zr-based MOFs and processability of the PVA-UiO-66 composite gels. Formation and properties of PVA-based composite gels formed from (a) NU-901, with 4,4′,4″,4‴-(1,3,6,8-pyrenetetrayl)tetrakis-benzoic acid) (H_4_TBAPy) linker; (b) UiO-67, with biphenyl-4,4′-dicarboxylic acid (BPDC) linker; and (c) MOF-525, with tetrakis(4-carboxyphenyl porphyrin) (TCPP) linker. For each formulation, we show the organic linker used in the synthesis, the crystal structures of each MOF obtained from Mercury software,^28^ composite gelation behavior via inversion tests, and grazing incidence X-ray diffraction (GIXD) patterns comparing the diffraction patterns of the MOFs formed in each composite gel to the simulated patterns for the corresponding MOFs. (d) Image showing that the PVA-UiO-66 composite gel thin film is free-standing. (e) Powder X-ray diffraction (PXRD) of the PVA-UiO-66 composite gel thin film. (f) Image showing that the PVA-UiO-66 composite gel thin film exhibits conformality. For the PVA-UiO-66 composite gel thin film, the PVA wt % is 2.5. Image of the NU-901 crystal structure is adapted with permission from ref (30). Copyright 2020 American Chemical Society. Image of the UiO-67 crystal structure is adapted with permission from ref (52). Copyright 2017 American Chemical Society. Image of the MOF-525 crystal structure is adapted with permission from ref (32). Copyright 2012 American Chemical Society.

To determine the generalizability of this composite gel formation process, we attempted to form composite gels with PVA and different Zr-oxo-based MOFs (NU-901 (Figure S14),^30^ UiO-67,^31^ and MOF-525^32^). We selected PVA as it facilitated the formation of most crystalline UiO-66 crystals within the composite gels among the polymers we studied. Just as in PVA-UiO-66 composite gel synthesis, we added a solution of Zr-oxo clusters to a solution containing PVA and the corresponding organic linker for each Zr-based MOF (Table 1).^23,33^ For MOFs containing dicarboxylic acid linkers, we used double the linker:Zr ratio used in the previous syntheses.^23^ For MOFs containing tetracarboxylic acid linkers, we used the same linker:Zr used in the previous syntheses.^33^ All formulations produced self-supporting gels, and SEM images (Figures S15–S17) and GIXD patterns confirmed the presence of each MOF within their respective composite gels (Figure 3a–c). The thermal stability observed in the PVA-UiO-66 composite gels persisted across the PVA-NU-901, PVA-UiO-67, and PVA-MOF-525 composite gels, which all passed the inversion test after being heated to 40 °C (Figure S18). Encouragingly, the examples presented here involving three different MOFs demonstrate the generalizability of this composite gel formation process.

**Table 1:** Organic Linkers Used in the Synthesis of PVA-Zr-MOFs Composite Gels

composite gel	organic linker used to obtain the composite gel	amount of organic linker used in composite gel synthesis	molar ratio of linker:Zr in the composite gel	molar ratio of linker:Zr in the literature
PVA-UiO-66^2^	H_2_BDC	120.0 mg	2:1	1:1
PVA-NU-901^33^	H_4_TBAPy	50.0 mg	1:5	1:5
PVA-UiO-67^23^	BPDC	175.0 mg	2:1	1:1
PVA-MOF-525^a^	TCPP	60.0 mg	1:5	

a Since MOF-525, like NU-901, contains a tetratropic organic linker, we used the same linker:Zr ratio for MOF-525 composites as for the PVA-NU-901 composites.

After preparing composites from different MOFs, we sought to demonstrate that this composite gel formation procedure could be extended to preparing conformable gel films from PVA-UiO-66 composites. We heated the composite gel to make it liquid and amenable to spin-coating. Spin-coating the composite onto glass substrates, followed by allowing the sample to set in ambient conditions for 24 h yields a free-standing gel film (Figure 3d). The thin film contains UiO-66 particles as demonstrated by the diffraction pattern (Figure 3e) and is capable of conforming to objects (e.g., a pencil as shown in Figure 3f), showcasing the enhanced processability imparted to MOFs by incorporation into these composites.

### Sorptive Capacity and Sustained Release of PVA-MOF Composite Gels

We next examined the sorptive properties of the composite gels relative to Zr-oxo-cross-linked polymer gels and MOF powder. For these experiments, we used composite and Zr-oxo-cross-linked PVA gels containing 3 wt % polymer, as they were easier to cut into uniform samples than the gels containing 2 wt % polymer. Rather than performing these studies in DMSO, we opted for aqueous conditions more relevant to drug delivery and environmental remediation applications. We first attempted to synthesize the PVA-UiO-66 composite gels in aqueous conditions, and while the white, opaque color of the solution suggested MOF formation, we did not observe gelation, likely due to the high concentration of water hydroxyl groups saturating the binding sites on the Zr-oxo clusters and outcompeting PVA hydroxyl groups (Figure S19). Therefore we synthesized composite gels in DMSO and to transition the gels from DMSO into aqueous solvent, we instead dialyzed the DMSO-swollen PVA-UiO-66 composite gels and controls (i.e., MOF powder and PVA-Zr-oxo gels) against RO water. We confirmed the complete removal of DMSO from the composite gels using ^1^H nuclear magnetic resonance spectroscopy (Figure S20 and Table S1). UiO-66 crystallinity was also maintained after solvent exchange, suggesting no disruption of UiO-66 structure during dialysis (Figure S21b). Since dialysis removes unincorporated organic linker and dissolved Zr-oxo clusters, we used thermal gravimetric analysis (TGA) to compare the compositions (i.e., wt % Zr) of the materials before and after solvent exchange (Figures S22–S25 and Table S2). The Zr content in the PVA-Zr-oxo control gels decreased substantially from 7.8 to 1.2 wt % Zr during solvent exchange, indicating that dialysis removes much of the Zr-oxo that is cross-linking polymer chains, and the resulting samples are primarily PVA hydrogels. In contrast, the PVA-UiO-66 composite gels retained more of their Zr during solvent exchange, only decreasing from 7.0 to 4.3 wt % Zr. Since the composite gels retain more Zr than the PVA-Zr-oxo control gels during solvent exchange, it is likely that the Zr-oxo clusters in the composites are primarily incorporated into UiO-66. Though we could not calculate the % Zr in the UiO-66 powder prior to solvent exchange, as it did not precipitate in DMSO, it contained an appreciable amount of Zr (21.3 wt %) after dialysis.

**Figure 4:**
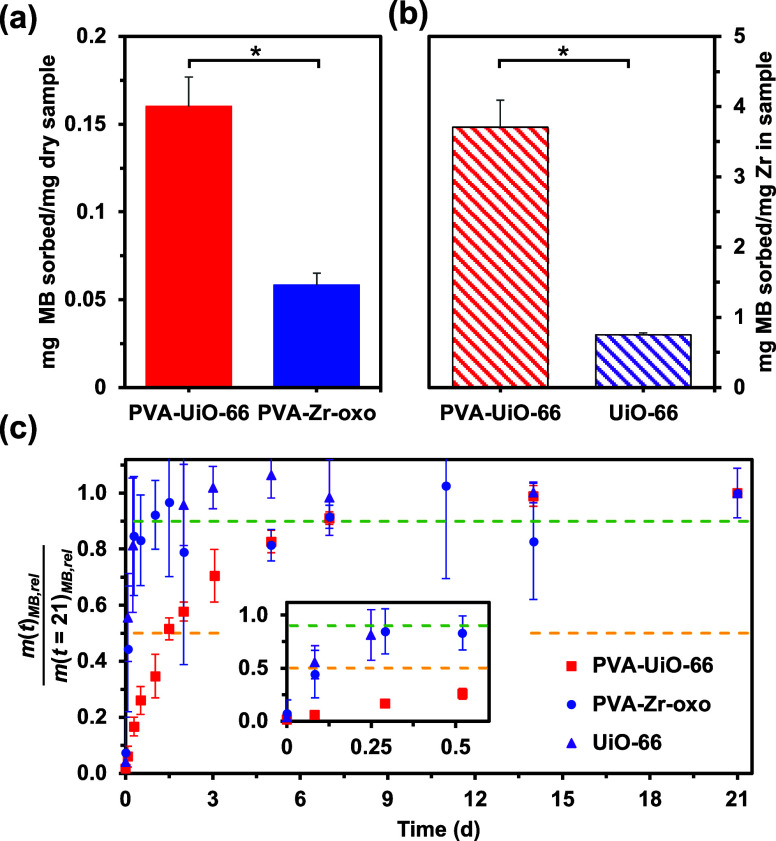
Sorptive capacity and release behavior of PVA-UiO-66 composite hydrogels (red) relative to that of PVA-Zr-oxo hydrogels (blue), and UiO-66 powder (purple) in ultrapure water. (a) Methylene blue (MB) sorption into PVA-UiO-66 composite hydrogels and PVA-Zr-oxo hydrogels after 7 days (mg MB/mg dry sorbent) shows UiO-66 incorporation into polymer increases MB sorption per mass of dry sorbent. (b) MB sorption into PVA-UiO-66 composite hydrogels and UiO-66 MOF (mg MB/mg Zr) after 7 days shows that forming UiO-66 in the presence of PVA increases MB sorption capacity on a per Zr basis. (c) Mass of MB released from each formulation at any given time (*m*(*t*)_MB,rel_) relative to the mass released after 21 d (*m*(*t* = 21)_MB,rel_), with the first 12 h (inset) highlighted. Yellow and green dashed lines denote release of 50% and 90% MB relative to that released at 21 days, respectively. While the PVA-Zr-oxo hydrogels (blue, circles) and UiO-66 powder (purple, triangles) display burst release behavior, releasing 90% of their cargo within less than 24 h, the PVA-UiO-66 composite hydrogels (red, squares) do not release 90% of the loaded MB until day 7. Error bars represent the standard deviation between three separately synthesized samples, with * in panels (a, b) indicating an equal variances two sample *t* test giving *p* < 0.05.

After each formulation was switched into aqueous solvent, we first monitored the sorption of the small molecule dye methylene blue (MB, 320 g/mol) into each by measuring the decrease in MB absorbance (660 nm) in the surrounding solution. We elected to study sorption of MB into these materials to compare their sorptive characteristics in the hydrated state. After 7 days, when sorption plateaued in the gel formulations and each sample was saturated with MB, we compared the amounts sorbed into each formulation (Figures S26–S29 and Table S3). The PVA-UiO-66 composite hydrogels sorbed significantly more MB (0.16 ± 0.02 mg MB/mg dry sample) than the PVA-Zr-oxo hydrogels (0.06 ± 0.01 mg MB/mg dry sample), indicating that the MOF contributes appreciable sorption capacity to the composites (Figure 4a). The increased MB sorption capacity of the composite gels relative to the PVA-Zr-oxo hydrogels, extended to composites prepared with all other Zr-oxo MOFs described in Figure 3 (PVA-MOF-525, PVA-UiO-67, and PVA-NU-901, all synthesized with 2 wt % PVA) (Figure S29).

We next determined how the polymer impacts the sorption properties of MOFs by comparing MB sorption into PVA-UiO-66 composite hydrogels relative to UiO-66 MOF powder alone in ultrapure water. On a per mass of sorbent basis, our PVA-UiO-66 composite hydrogels MB sorptive capacities were on par with state-of-the art reported values for UiO-66 (Table S4).^34,35^ However, rather than comparing these on a per mass of sorbent basis, we compared the samples on a per mass Zr basis to isolate the role of the polymer. On a per g of Zr basis, the composite hydrogels sorbed substantially more MB (3.71 ± 0.39 mg MB/mg Zr) than UiO-66 (0.75 ± 0.03 mg MB/mg Zr) after 7 days (Figure 4b and Table S3). We suspect that forming UiO-66 in the presence of PVA disperses the UiO-66 particles within the hydrogel composite, leading to a greater UiO-66 surface area in the composite gels and the higher sorption per Zr relative to that of UiO-66 formed in the absence of polymer. Taken together, these sorptive studies highlight how forming MOFs in the presence of polymer boosts the sorption capacity relative to polymer networks without MOF, and also relative to MOF formed in the absence of polymer.

We next studied the release of MB from the composite gels relative to the PVA-Zr-oxo and UiO-66 control samples in ultrapure water. After allowing MB to load into each sample for 7 days, we replaced the surrounding solution with an equivalent volume of DI water and monitored the release of MB into the surrounding solution. We report the mass of MB released into the outer solution (*m*(*t*)_MB,rel_) relative to the total amount released after 21 d (*m*(*t* = 21)_MB,rel_), specifically focusing on the time taken for each formulation to release 50% and 90% of their respective *m*(*t* = 21)_MB,rel_ (Figure 4c). While the PVA-Zr-oxo hydrogel and UiO-66 controls exhibited burst release behavior, releasing 50% and 90% of their respective *m*(*t* = 21)_MB,rel_ in less than 3 and 24 h, respectively, the PVA-UiO-66 composite hydrogels showed a sustained MB release profile, releasing 50% and 90% of *m*(*t* = 21)_MB,rel_ in 1.5 and 7 d, respectively. While there was a particularly large amount of error between the PVA-Zr-oxo samples, we ascribe this to the relatively low amounts of MB released into the solution, with MB concentrations approaching the limit of reliable absorbance detection of the instrument. Comparing the release profiles suggests that the combination of UiO-66 and PVA slows solute diffusion relative to their components.

After demonstrating the sorption and release of MB, we investigated the encapsulation and release of larger, therapeutically relevant cargo (*e.g.,* peptides). Despite their high potency, high selectivity, and low toxicity, therapeutic peptides^36−38^ are often rapidly cleared from circulation by proteolytic degradation and renal clearance, leading to poor efficacy.^39^ Angiotensin 1–7 (DRVYIHP, Ang 1–7), a therapeutic peptide with antitumor and cardioprotective properties, has an *in vivo* half-life of just 30 min.^40,41^ Encapsulation and sustained release into a carrier such as polymer-MOF composite hydrogels could offer a way to extend the half-life of Ang1–7. Because Ang 1–7 (899 g/mol) (Figure S30) is larger than MB (320 g/mol) we first attempted to encapsulate it within UiO-67 and MOF-525, which have larger pores (12 and 18 Å, respectively) than UiO-66 (6 Å).^31,32^ Though reverse-phase high-performance liquid chromatography showed little decrease in Ang 1–7 absorbance in the outer solution after incubation with UiO-67 after 27 h, there was no detectable trace of Ang 1–7 in the solution containing MOF-525, suggesting that Ang 1–7 sorbed into MOF-525, but not UiO-67 (Figure S31). Therefore, we performed the Ang 1–7 encapsulation and release experiments using PVA-MOF-525 composite hydrogels. We also confirmed total removal of DMSO and that MOF-525 crystallinity is maintained in the PVA-MOF-525 composite hydrogels after dialysis (Figures S20 and S21).

**Figure 5:**
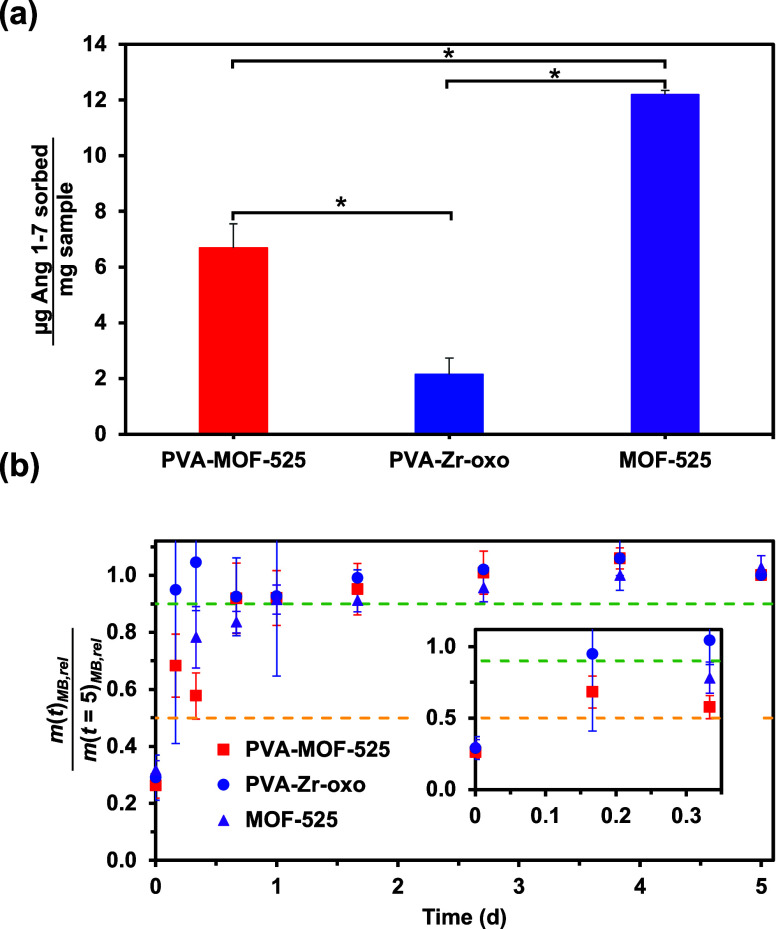
Sorptive capacity and release behavior of PVA-MOF-525 composite hydrogels (red), PVA-Zr-oxo hydrogels (blue), and MOF-525 powder (purple) for the therapeutic peptide Ang 1–7 in ultrapure water. (a) Mass of Ang 1–7 (μg) sorbed into each sample after 7 days, normalized relative to the mass of each sample (mg), showing more Ang 1–7 to sorb into the MOF-525 powder than into the PVA-Zr-oxo and PVA-MOF-525 composite hydrogels on a per mass basis. (b) Mass of Ang 1–7 released from each formulation at any given time (*m*(*t*)_Ang 1-7,rel_) relative to the mass released after 5 d (*m*(*t =* 5)_Ang 1-7,rel_), with the first 6 h (inset, bottom right) highlighted. Yellow and green dashed lines denote release of 50% and 90% Ang 1-7 relative to that released at 5 days, respectively. The PVA-MOF-525 composite hydrogels (red, squares), PVA-Zr-oxo hydrogels (blue, circles), and MOF-525 powder (purple, triangles) all display burst release behavior, releasing 90% of their cargo within less than 24 h. Error bars represent the standard deviation between three separately synthesized samples, with * in panel (a) indicating an equal variances two sample *t* test giving *p* < 0.05.

To encapsulate Ang 1–7 into PVA-MOF-525 composite hydrogels, PVA-Zr-oxo hydrogels, and MOF-525 powder, we incubated each sample in a solution of Ang 1–7 in ultrapure water. Here, we normalized the sorption data by the overall weight of each sample (mg Ang 1–7 sorbed/mg material). After 7 days, when the samples were found to be saturated (Figure S32), the MOF-525 powder sorbed significantly more Ang 1–7 (12.2 ± 0.2 μg Ang 1–7/mg sample) than both the PVA-Zr-oxo (2.2 ± 0.6 μg Ang 1–7/mg sample) and PVA-MOF-525 composite hydrogels (6.7 ± 0.9 μg Ang 1–7/mg dry sample) (Figure 5a and Table S5). On a dry weight basis, the PVA-MOF-525 composite hydrogel sorbed significantly more Ang 1–7 (79.7 ± 10.2 μg Ang 1–7/mg dry sample) than the PVA-Zr-oxo hydrogel (45.3 ± 12.5 μg Ang 1–7/mg dry sample) and MOF-525 powder (12.2 ± 0.2 μg Ang 1–7/mg dry sample) (Figure S33 and Table S5). The release profiles of the 3 formulations in ultrapure water, however, were fast and indistinguishable, with all formulations plateauing in less than 1 day (Figure 5b). The burst release observed in the PVA-MOF-525 composite gels could be due to the larger pore size of MOF-525; however, given that the PVA-MOF-525 composite gel only released 29% ± 3% of the encapsulated Ang 1–7 (Figure S34 and Table S6), it is also possible that the Ang 1–7, which has 2 carboxylic acids, is interacting with the open Zr-oxo sites, and these interactions prevent any bound peptide from being released. While future optimization of PVA-MOF composite hydrogels for larger peptide cargo is still needed, however, the enhanced sorption capacity of the composite hydrogels for Ang1–7 relative to the PVA-Zr-oxo hydrogel and MOF-525 controls is certainly encouraging.

## Conclusions

In this work, we found that polymer molecular details dictate the formation and properties of polymer-MOF composite gels prepared by forming MOF in the presence of the polymeric component. While we found that polymers with functional groups that bind the Zr-oxo clusters of MOFs without outcompeting the organic linkers allowed for concurrent gelation and MOF formation, our experiments with PEG suggest that polymer entrapment within MOFs by alone is sufficient to facilitate gelation within composites, expanding the polymeric options available for future polymer-MOF composite formulations. Through simulations, we found that both chemical interactions between polymers and metal clusters as well as physical entrapment of polymers within MOF pores play a role in polymer-MOF composite gel formation. Composite gel formation with various PVA-Zr-based MOF composite gels demonstrated the generalizability of the composite gel formation technique to other MOFs, and the fabrication of films suggests the ability to extend this composite formation process to conformable gel films. PVA-UiO-66 composite hydrogels demonstrated higher sorption and sustained release of MB relative to UiO-66 powder and/or PVA-Zr-oxo hydrogels, showcasing the composites as beneficial materials for various sorption applications. While forming MOF-525 in the presence of PVA yielded increased sorptive capacity relative to the metal cross-linked PVA hydrogels, the sorptive capacity of the PVA-MOF-525 composite hydrogels (8 wt %) was significantly lower than those reported for insulin, another therapeutic peptide, into either MIL-100, NU-1000, or MOF-545 (34–63 wt %).^42^ Further, unlike the PVA-UiO-66 composites, we observed a burst release of Ang 1–7 from the PVA-MOF-25 composite gels. To that end, we expect both sorptive capacity and release profiles to highly depend on both the solute and the adsorbent being used, and we look forward to exploring different combinations of MOFs and polymers to optimize these properties for larger peptide cargo. By understanding the role of molecular details of polymers and MOFs on the properties of composite gels prepared by forming MOFs in the presence of polymers, this study expands the design space of polymer-MOF gels, and offers a method to increase the processability and performance of MOFs for applications ranging from separations to medicine.

## Materials and Methods

### Materials

Zirconium(IV) propoxide solution (70 wt % in 1-propanol, Zr(O*n*Pr)_4_), *N*,*N*-dimethylformamide (DMF, ≥ 99.8%), poly(vinyl alcohol) (PVA: *M*_w_ ∼146000–186000 g/mol and 99%+ hydrolyzed; *M*_w_ ∼31000–50000 g/mol and 98–99% hydrolyzed; and *M*_w_ ∼9000–10000 g/mol and 80% hydrolyzed), poly(ethylene glycol) (PEG, *M*_v_ ∼100000 g/mol), poly(acrylic acid) (PAA, *M*_w_ ∼1033000 g/mol), terephthalic acid (H_2_BDC, 98%), 2-aminoterephthalic acid (H_2_ATA, 99%), biphenyl-4,4′-dicarboxylic acid (BPDC, 97%), 4,4′,4″,4‴-(porphine-5,10,15,20-tetrayl)tetrakis(benzoic acid) (TCPP, dye content 75%), dimethyl sulfoxide-d6 (deuteration ≥99.8%), acetic acid (≥99.7%), 4-ethoxycarbonylphenylboronic acid (95%), dioxane (99%), 1,3,6,8-tetrabromopyrene (97%), potassium phosphate tribasic (98%), tetrakis(triphenylphosphine)palladium(0) (99%), potassium hydroxide (90%), chloroform (99%), dichloromethane (99.8%), methylene blue (certified by the Biological Stain Commission, dye content ≥82%) diisopropyl carbodiimide (DIC, 99.8%), *N,N′*-dimethylformamide (DMF, 99%), Oxyma Pure (99%), piperidine (99%), *N,N*-Diisopropylethylamine (DIPEA, 99%), trifluoroacetic acid (TFA, 99%), triisopropyl silane (TIPS) (98%), 2,2′-(ethylenedioxy)diethanethiol (DODT) (95%), diethyl ether (99%), and acetonitrile (ACN, HPLC grade, 99.8%) were purchased from Sigma-Aldrich. Dimethyl sulfoxide (DMSO, ≥ 99.9%), methanol (99.9%), and hydrochloric acid (HCl, 36.5–38%) were purchased from Fisher Scientific. Potassium iodide (KI, > 99%) was purchased from VWR. Poly(acrylamide-*co*-acrylic acid) (PAAA, AMD: AA = 9:1, *M*_w_ ∼210000 g/mol) was purchased from Polymer Source Inc. Fluorenylmethoxycarbonyl(Fmoc)-protected amino acids and 2-chlorotrityl chloride resin (0.6 mmol/g) were purchased from Advanced ChemTech (Louisville, Kentucky). 4,4′,4″,4‴-(pyrene-1,3,6,8-tetrayl)tetrabenzoic acid (H_4_TBAPy), the organic linker for NU-901, was synthesized following a previously published procedure, and characterized using ^1^H nuclear magnetic resonance spectroscopy (Figure S14).^43^ All water was purified by in-house reverse osmosis (RO). Ultrapure water refers to water purified by a Thermo Scientific Barnstead Smart2Pure water purification system (18.2 mΩ·cm).

### Synthesis of Zr-oxo Clusters in DMSO

Synthesis conditions were adapted from a previously published procedure.^23^ Briefly, in a 20 mL glass vial, Zr(O*n*Pr)_4_ (355 μL, 0.792 mmol, stored under *N*_2_(*g*) until use) and acetic acid (4.00 mL, 69.9 mmol) were added to solvent (DMSO, 7.00 mL). The solution was sonicated for 10 min, placed in an oven at 130 °C for 2 h, and cooled to room temperature to yield Zr-oxo clusters for use in further synthesis.

### Synthesis of Zr-oxo Cluster in DI Water

Synthesis conditions were adapted from ref (24). In a 20 mL glass vial, ZrOCl_2_.8H2O (1.288 g) and acetic acid (5.00 mL) were dissolved in DI water (12.00 mL) via bath sonication. Then, the solution was heated at 70 °C for 2 h, and cooled to room temperature to yield Zr-oxo clusters for use in further synthesis.

### Synthesis of UiO-66 Powder in DMSO

Into a Zr-oxo cluster solution in DMSO (10.0 mL, 0.697 mmol of Zr), H_2_BDC (120.0 mg, 0.722 mmol) was added and dissolved using sonication. The solution was stirred for 24 h at room temperature. The particles produced from this synthesis were not isolatable by centrifugation, and therefore were isolated by dialysis against deionized water in a 3.5 kDa molecular weight cutoff regenerated cellulose dialysis membrane (Spectra/Por). Each water change was allowed to equilibrate for ≥3 h. After dialysis, the UiO-66 particles were allowed to settle in DI water and the sediment was dried at room temperature for 48 h in a fume hood to obtain the dried UiO-66 powder.

### Synthesis of UiO-67 Powder in DMSO

Into a Zr-oxo cluster solution in DMSO (10.0 mL, 0.697 mmol of Zr), BPDC (175.0 mg, 0.722 mmol) was added and sonicated for 10 min. The solution was heated at 130 °C for 24 h. Then the UiO-67 was separated from the supernatant by centrifugation (10 min, 9000*g*, 4 °C), and washed three times with DMSO and three times with acetone by centrifugation (10 min, 9000*g*, 4 °C). The UiO-67 sample was dried at 80 °C for 24 h to obtain the dried UiO-67 powder.

### Synthesis of MOF-525 Powder in DMSO

Into a Zr-oxo cluster solution in DMSO (10.0 mL, 0.697 mmol of Zr), TCPP (60.0 mg, 0.076 mmol) was added and sonicated for 10 min. The solution was heated at 130 °C for 24 h. Then the MOF-525 was separated from the supernatant by centrifugation (10 min, 9000*g*, 4 °C), and washed three times with DMSO and three times with acetone by centrifugation (10 min, 9000*g*, 4 °C). The MOF-525 sample was dried at 80 °C for 24 h to obtain the dried MOF-525 powder.

### Synthesis of Polymer-UiO-66 Composite Gels

In a 20 mL scintillation vial, 220.0 mg of polymer (PAA, PAAA, PVA, or PEO) was dissolved in DMSO (5.00 mL) by stirring at 120 °C for up to 45 min, then cooling to room temperature, where the polymers remained soluble. H_2_BDC (120.0 mg, 0.722 mmol) was then added to the polymer solution. Next, the Zr-oxo cluster solution (5.00 mL in DMSO, 0.349 mmol of Zr) was added to the polymer-H_2_BDC solution (5.00 mL in DMSO) and the mixture was shaken for 1 min and allowed to stand at room temperature for 24 h. After 24 h, the gelation of the mixture was evaluated using an inversion test (i.e., inverting the vial). The polymer-UiO-66 composite gels with different wt % of polymer were synthesized by varying the polymer amount in a constant volume of DMSO (5.00 mL). The gels were stored at ambient temperature until further use.

### Synthesis of Polymer-Zr-oxo Gels

In a 20 mL scintillation vial, 220.0 mg of polymer (PAA, PAAA, PVA, or PEO) was dissolved in DMSO (5.00 mL) by stirring at 120 °C for up to 45 min, then cooling to room temperature, where it remained soluble. Zr-oxo cluster solution (5.00 mL in DMSO, 0.349 mmol of Zr) was added to the polymer solution (5.00 mL in DMSO) and the mixture was shaken for 1 min and allowed to stand at room temperature for 24 h. After 24 h, the gelation of the mixture was evaluated using an inversion test. The gels were stored at ambient temperature until further use.

### Synthesis of Polymer-UiO-66 Physical Mixture

In a 20 mL scintillation vial, 220.0 mg of polymer (PAA, PAAA, PVA, or PEO) was dissolved in DMSO (5.00 mL) by stirring at 120 °C for up to 45 min, then cooling to room temperature, where it remained soluble. UiO-66 powder (95.0 mg, synthesized in DMSO) was added to the polymer solution (5.00 mL in DMSO) and the mixture was shaken for 1 min and allowed to stand at room temperature for 24 h. After 24 h, the gelation of the mixture was evaluated using an inversion test. These mixtures were stored at ambient temperature until use for characterization.

### Synthesis of PVA-Zr-MOFs Composite Gels

In a 20 mL scintillation vial, 220.0 mg of PVA was dissolved in DMSO (5.00 mL) by stirring at 120 °C for up to 45 min, then cooling to room temperature, where it remained soluble. Organic linkers (Table 1) were then added to the PVA solution. Next, the Zr-oxo clusters solution (5.00 mL in DMSO, 0.349 mmol of Zr) was added to PVA-linker solution (5.00 mL) and the mixture was shaken for 1 min and allowed to stand at room temperature for 24 h, after which the gelation of the mixture was evaluated using an inversion test. The linker amount was determined based on the molar ratio of linker:Zr used in the previously published syntheses to make MOFs (Table 1). The composite gels were stored at ambient temperature until further use.

### Synthesis of PVA-UiO-66 Composite Gel Film

**Scheme 1:**
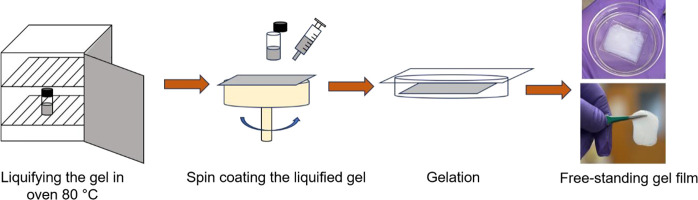
Synthesis Procedure to Process the PVA-UiO-66 Composite Gel into a Film

The PVA-UiO-66 composite gel film synthesis procedure has been illustrated in Scheme 1. First, a PVA-UiO-66 composite gel (2.5 wt %) in a vial was heated in the oven at 80 °C for 2 h. Due to heating, the gel yielded a viscous liquid which was removed via syringe (0.5 mL) and spin coated on a glass substrate at 150 rpm for 30 s. The spin coated liquid film was then left covered in a Petri dish overnight to yield a free-standing gel film. To record the diffraction pattern of the gel film, it was left uncovered to dry at room temperature for 3 d. Afterward, we recorded the diffraction of the dried gel film.

### Synthesis Trial of PVA-UiO-66 Composite in RO Water

In a 20 mL glass vial, 220.0 mg of PVA (*M*_w_ ∼146,000–186,000 g/mol) was dissolved in RO water (5.00 mL) by stirring at 130 °C for up to 120 min, then cooling to room temperature, where the polymer remained soluble. H_2_BDC (181.0 mg) and NaOH (80.0 mg) were added to the PVA solution. Next, Zr-oxo cluster solution (5.00 mL in RO water) was added to the polymer-H_2_BDC solution (5.00 mL in RO water) and the mixture was shaken for 1 min and allowed to stand at room temperature for 24 h. After 24 h, the gelation of the mixture was evaluated using an inversion test (i.e., inverting the vial).

### Synthesis of Angiotensin 1–7

Angiotensin 1–7 (DRVYIHP) was prepared using Fmoc-solid phase peptide synthesis with a CEM Liberty Blue microwave peptide synthesizer. To prepare the peptide with a carboxylic acid C-terminus, 2-chlorotrityl chloride resin (0.6 mmol/g) was used, and the entire synthesis was conducted at 25 °C to prevent premature cleavage of the ester bond connecting the peptide to the resin, which was observed above 50 °C. DIC (1 M in DMF) and Oxyma Pure (1 M in DMF) were used to mediate amino acid coupling, except for the first amino acid, which was coupled to the resin using KI (0.125 M in DMF) and DIPEA (1 M in DMF). 20% piperidine in DMF (v/v) was used to deprotect Fmoc groups preceding amino acid additions. After synthesis, the peptide was cleaved from the resin using a deprotection cocktail comprised of 92.5% TFA, 2.5% RO water, 2.5% TIPS and 2.5% DODT by volume for 3 h at room temperature under constant stirring. Following deprotection, the peptide solution was separated from the resin by gravity filtration, and the peptide solution was precipitate into cold diethyl ether (5–8 mL of peptide solution in 30 mL ether), and centrifuged (5 min, 2420*g*, 4 °C) using a Thermo Scientific Haraeus Multifuge X3R. The supernatant was then decanted and the precipitated peptide pellet was washed again with the same volume diethyl ether and isolated by centrifugation under the same conditions and the supernatant decanted. The peptide pellet was dried under vacuum for 1 h, dissolved in 5% ACN in ultrapure water (v/v) and frozen with liquid nitrogen immediately prior to lyophilization for 48 h to produce a fluffy cake that was easy to manipulate. 60–70% yield was achieved for each synthesis.

### Reverse-Phase High-Performance Liquid Chromatography (RP-HPLC)

Analytical RP-HPLC was performed at 35 °C with a flow rate of 1 mL/min on a Waters e2695 Alliance Separations Module, equipped with a XBridge C18 chromatographic separation column (4.6 mm × 50 mm, 3.5 μm beads) and a photodiode array detector (Waters 2489 UV/Visible). The mobile phase consisted of ultrapurified water and ACN, both with 0.1% TFA by volume for pH maintenance. Peptide purification was completed using preparative scale RP-HPLC, which was performed at 25.52 mL/min at room temperature on a Waters Empower system, equipped with a XBridge Prep C18 optimum bed density chromatographic separation column (30 mm × 150 mm, 5 μm beads) and a photodiode array detector (Waters 2489 UV/Visible). UV absorbance was monitored at 214 nm for both systems. Mobile phase gradients for Ang 1–7 on each system are detailed in Table 2. The preparative scale mobile phase gradients were derived from those used at the analytical scale using the Waters Gradient Chromatography Calculator online tool.

**Table 2:** RP-HPLC Mobile Phase Gradients

**time (m)**		**gradient**	
**analytical**	**preparative**	**% water +0.1% TFA (A)**	**% ACN + 0.1% TFA (B)**
0	0	95	5
0.5	2.22	82	18
3.5	15.07	80	20
3.7	15.93	5	95
4.2	18.07	5	95
4.8	20.65	95	5
6.0	25.79	95	5

### Electrospray Ionization Mass Spectrometry (ESI)

For mass spectrometry, Ang 1–7 was dissolved at a concentration of 50 μg/mL in DI water. The experiment was performed on an Agilent G7104C LC system equipped with an Agilent G1958–65268 Dual AJS electrospray ionization source and Agilent 6545B QTOF mass spectrometer. The sample (0.2 μL) was eluted on a linear gradient of 0.1% formic acid in water and 0.1% formic acid in methanol, from 5% methanol at 0 min to 100% methanol at 3 min. Separation was achieved using an Agilent InfinityLab Poroshell 120 EC-C18 (3.0 mm ID × 100 mm, 2.7 μm pore size). Mass spectrometry data was collected in negative ionization mode with the following ESI parameters: capillary voltage = 4000 V; nozzle voltage = 250 V; fragmentor voltage = 90 V; drying gas flow = 5 L/min; drying gas temperature = 325 °C; and nebulizer gas pressure = 60 psig.

### Solvent Exchange from DMSO to Water

All samples were placed in 3.5 kDa molecular weight cutoff regenerated cellulose dialysis membranes (Spectra/Por 7) containing 15–20 mL of water and dialyzed against RO H_2_O. After allowing the solution to equilibrate for ≥3 h, the dialysate was replaced with fresh RO H_2_O. After four total dialysate replacements, the samples were removed and stored in RO H_2_O at room temperature prior to use.

### Encapsulating Solutes into MOF-Based Carriers

To measure the mass (*m*, in mg) of a solute, *s* (either methylene blue (MB) or the peptide Angiotensin 1–7 (Ang 1–7)), encapsulated into the MOF-based carriers at any given time, *t,* or *m*(*t*)_*s*, carrier_, we first prepared an aqueous solution of solute *s* (0.05 mg/mL for MB or 0.57 mg/mL for Ang 1–7). This solution was termed the “outer solution”, os, and each sample carrier was added to a specific volume, *V*_os_ (mL). For consistency, we added 70–80 mg of each gel or 10–12 mg of UiO-66 powder to 17.5–20 mL of MB solution (4 mg gel/mL MB solution, 0.16 mg UiO-66/mL MB solution), and ∼100–120 mg gels and/or MOF-525 to 10–12 mL of Ang 1–7 solution (10 mg carrier/mL Ang 1–7 solution) for the encapsulation experiments. To calculate the sorption of a given solute *s* into each sample at time *t*, we started with a mass balance on the solute in the vial. Specifically, we considered *m*(*t*)_*s*, carrier_ would be the difference between the total mass of *s* in the vial at time *t* (*m*(*t*)_*s*, vial_) and that in the outer solution at time *t* (*m*(*t*)_*s*, os_) (eq 1).
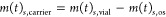
1

Rewriting eq 1 in terms of the concentration (*c*, in mM) of *s* in the outer solution gives eq 2.
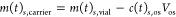
2

In eq 2, we assume *V*_os_ to be constant, as the carriers are swollen when they enter the vial and therefore are unlikely to absorb appreciable amounts water from the outer solution upon incubation. As we are measuring the absorbance of the outer solution (*A*(*t*)*_s_*_,os_) at the absorbance maximum wavelength (λ) specific to *s* (660 nm for MB and 277 nm for Ang 1–7), we convert it to a corresponding concentration (*c*(*t*)_*s*, os_) using the Beer–Lambert law (eq 3),
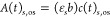
3where ε_*s*_ is the molar absorptivity of *s* and *b* is the optical path length in the configuration used (in this case a Biotek Synergy 4 plate reader), which we lump into one term determined from calibration curves for MB (Figure S26a) and Ang 1–7 (Figure S26b), fitting the data linearly to determine the (ε_*s*_*b*) term for each solute *s* (5 to 6× 10^–3^ absorbance units/μM MB and 7 × 10^–4^ absorbance units/μM Ang 1–7). To account for absorbance due to the carrier rather than to *s* (e.g., linker that leaches into the outer solution), we placed each sample in two aqueous solutions, one containing *s* and the other containing only RO water. Once we subtracted the absorbance of water alone, which was often negligible relative to the absorbances of *s*, from that measured for each sample, we accounted for any absorbance from the carrier at λ by subtracting the absorbance of the control (cntrl) sample outer solution (*A*(*t*)_cntrl,os_) from that of the solution containing *s* (*A*(*t*)_s,os_) to calculate a corrected absorbance of *s* in the outer solution (A(t)^*^_s__*,os*_) (eq 4).
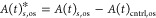
4

Using our calibration curve for *s*, we then converted *A*(*t*)^*^_s,os_ to c(*t*)_*s*, os_ (eq 3), and solved for *m*(*t*)_*s*, os_ (eq 5).
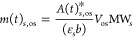
5where MW_*s*_ is the molecular weight of *s*. To measure *A*(*t*)^*^_s,os_, we removed an aliquot (with a volume *V*_a_) of the outer solution at each time point (*V*_a_ = 0.6 mL for MB and *V*_a_ = 0.1 mL for Ang 1–7); however, removing these aliquots also *m*(*t*)_*s*, vial_. To accurately calculate *m*(*t*)_*s*, vial_, we need to calculate the mass of *s* in each aliquot (*m*(*t*)_*s*, aliquot_) that we are removing from the vial using (eq 6).
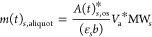
6

After every aliquot, we replaced the volume removed in each aliquot (*V*_a_) with the same volume of DI water (not containing *s*) to keep *V*_os_ constant. We then calculated *m*(*t*)_*s*, vial_ by subtracting the sum of the mass of the aliquots removed before the time t () from the initial mass of *s* added to the vial (*m*(0)_s, vial_) (eq 7).
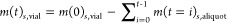
7

As *s* is only present in the outer solution at *t* = 0, *m*(0)_*s*,vial_ = *m*(0)_*s*, os_, and we can rewrite eq 7 as eq 8.
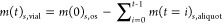
8

Rewriting eq 8 in terms of absorbance gives us a mass balance on the vial in terms of measured and/or known variables (eq 9).
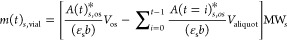
9

As we now know *m*(*t*)_*s*, os_ and *m*(*t*)_*s*, vial_ in terms of measured values, we can rearrange eq 1 to solve for *m*(*t*)_*s*, carrier_ using the *A*(*t*)^*^_s,os_ in the outer solution (eq 10).
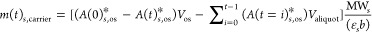
10

After calculating *m*(*t*)_*s,* carrier_, we normalized these values relative to either the total weight of the carrier, dry weight of the carrier, or the weight of Zr in the carrier, as indicated. To calculate the dry weight of the carriers we weighed one sample of each of the hydrogels and MOF powder used in the encapsulation experiments and dried it at room temperature for 24 h. We then weighed the mass of the dried carriers and calculated a dry/wet weight ratio for each sample (dry weight of hydrogel/wet weight of hydrogel). The dry mass of each hydrogel used in the encapsulation experiment was calculated by multiplying the wet weight of the gel by this ratio, assuming similar swelling ratios across each of the hydrogel samples. As the MOF powder was added into each vial as a dry solid, its dry:wet mass ratio was 1. The Zr weight of each gel was calculated by multiplying the dry weight of each hydrogel or MOF powder used for encapsulation by the Zr wt %, as determined by TGA (Figures S22–S25 and Table S2).

### Release of Solutes from MOF-Based Carriers

After encapsulation was complete, the outer solution of each sample (*s* and *cntrl*) was pipetted out and replaced with an equal amount of RO water. To account for the lower molar absorptivity of Ang 1–7 at 277 nm relative to MB at 660 nm, the outer solution was replaced with half of the volume of RO water (20 mg carrier/mL) in the Ang 1–7 experiments to increase *A*(*t*)_^*^Ang 1–7,os_. In these experiments, we were interested in determining the mass of solute released into the outer solution as a function of time, or *m*(*t*)_*s,* rel_. To determine *c*(*t*)_*s*, os_, we measured *A*(*t*)^*^_s,os_ at each time point *t*. We can directly measure *m*(*t*)_s,rel_ through eq 5; however, we still have to account for *m*_*s*,aliquot_, as we are removing a mass of *s* from our system for each measurement. Taken together, we can use eq 11 to calculate *m*(*t*)_*s*_,_rel_ from a measured *A*(*t*)^*^_s,os_.
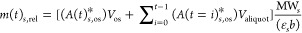
11

Here, we report the mass of *s* released into the outer solution (*m*(*t*)_MB,rel_) relative to the total amount of MB released from the carriers after 21 d (*m*(*t* = 21)_MB, rel_) and the total amount of Ang 1–7 released from the carriers after 5 d (*m*(*t* = *5*)_Ang 1–7, rel_) to better visualize and compare differences in the release profiles over time between each sample.

### Statistics for Encapsulation and Release Experiments

Each measurement represents an average of three independently synthesized samples, meaning 3 *s* and 3 *cntrl* samples from separate synthetic batches. Error bars represent the standard deviation between these three measurements. Each absorbance measurement represents the average of three individual measurements of one sample, and the standard deviation between these measurements was negligible.

### Plate Reader Absorbance Measurements

The absorbance of MB and Ang 1–7 were measured at 660 and 277 nm, respectively, on a Biotek Synergy 4 plate reader. All measurements were conducted in RO H_2_O. Calibration curves were developed in triplicate through serial dilution of the MB and Ang 1–7 stock solutions.

### Simulation Methods

Molecular dynamics (MD) simulations were performed using the large-scale atomic/molecular massively parallel simulator (LAMMPS)^44^ in an NVT ensemble with a time step of 1 fs at 300 K. Lammps-interface^45^ was used to prepare the LAMMPS data file with universal force field (UFF)^46^ parameters. A 2 × 2 × 2 supercell of UiO-66 was used to support a nonbonded interaction cutoff of 12.5 Å. Nonbonded interactions were modeled by a Lennard-Jones potential and cross-terms were calculated using Lorentz–Berthelot mixing rules.

Adaptive biasing force method (ABF)^47^ as implemented in Software Suite for Advanced General Ensemble Simulations (SSAGES)^48^ was used to estimate the free energy barrier of different monomer mimics of polyethylene glycol (PEG, CH_3_OCH_2_CH_3_) and poly(vinyl alcohol) (PVA, CH_3_CH_2_OHCH_3_), traversing through UiO-66. We used the monomer mimic center of mass (COM) as a collective variable in the *y* and *z* dimension and a bin size of 0.2 Å to generate a 2D grid to estimate the free energy profile. We obtained the free energy profile in the *y* and *z* dimensions and display the average value of those in the *x* direction.

The finite temperature string (FTS) method^49^ as implemented in SSAGES was used to determine the traversing pathway for different monomers through the pores of UiO-66. The final pathway obtained from FTS depends on the global features of the free energy landscape. In our study, an initial string was generated from our MD simulations that traces a path from a small pore to a large pore in UiO-66 using the 2D free energy profile to guide our initial selection. We then discretized the string into equally spaced nodes, which represent the COM of the monomer mimics. Each node explores a Voronoi cell associated with it. After a prescribed number of steps, the running averages of the variables of interest are updated i.e., the position of the monomer mimic COM. The string is then updated toward the running averages while also guaranteeing that the nodes on the curve are an equal arc length apart and that the curve is smooth (usually using a cubic spline). This process is repeated until the string converges.

We interpolate the converged string points and then use the *y* and *z* coordinates of the nodes to map it to the 2D free energy profile obtained from ABF simulation to extract the free energy profile of PEO and PVA traversing through UiO-66. The pore limiting diameter (PLD) and largest cavity diameter (LCD) estimated using PoreBlazer^50^ for UiO-66 are 3.40 and 8.06 Å, respectively. The estimated effective diameters for PEO and PVA are 2.60 and 4.83 Å, respectively, calculated by the taking the average of length and width of the monomers as shown in Figure 2d.

### Grazing Incidence X-ray Diffraction (GIXD)

GIXD experiments were performed at beamline 11–3 of the Stanford Synchrotron Radiation Lightsource at SLAC National Accelerator Laboratory with a fixed beam energy of 12.7 keV. The two-dimensional (2D) GIXD diffraction patterns were recorded using a Rayonix MX225 CCD area detector. Wet gel samples and dry UiO-66 powder were put on a metal substrate holder to collect the 2D diffraction patterns. The sample-to-detector distance was 316 mm. Fast Azimuthal Integration (pyFAI) using python was used to obtain one-dimensional (1D) diffraction patterns from 2D GIXD diffraction patterns.

### Coherence Length Calculation

Coherence length was calculated using the Scherrer equation^26^ (eq 12):
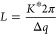
12where *L* is coherence length, Δ*q* is the full width at half-maximum (fwhm) of the most intense peak in the diffraction pattern, and *K* is a dimensionless shape factor that depends on the shape of the crystallites. For spherical particles, *K* = 0.93. All calculated coherence lengths of UiO-66 particles synthesized in the presence of polymers are reported in Table 3.

**Table 3:** Coherence Length of UiO-66 Particles Synthesized in the Presence of Polymers in DMSO

**sample name**	**coherence length (nm)**
PVA-UiO-66 gel	44
PEG-UiO-66 gel	32
PAAA-UiO-66 gel	26

### Powder X-ray Diffraction (PXRD) for Analysis of the Gel Film

To determine the presence of UiO-66 within the composite gel film spun on a glass substrate, PXRD patterns were collected using an Empyrean multipurpose X-ray diffractometer. Before the PXRD analysis, the gel film was dried at room temperature for 72 h. X-rays were generated using a water-cooled sealed X-ray tube with line focus Cu-anode (λ = 1.54 Å) operating at 45 keV and 40 mA. The one-dimensional (1D) PXRD diffraction patterns were recorded using a GaliPIX3D detector.

### Scanning Electron Microscopy-Energy Dispersive X-ray Spectroscopy (SEM-EDS)

The gels were dried at room temperature on a glass slide prior to characterization. The dry gel was sputter coated with a layer of Au/Pd using a Gatan 682 Precision Etching and Polishing System (PECS). SEM Images of the dry gel were obtained using a FEI quanta 650 field-emission secondary electron microscope. The accelerating voltage of the primary beam was kept between 5 kV and 15 kV, and the spot size was kept below 4. EDS analysis was performed using the same instrument at the voltage between 10–15 kV and spot size at 4.

### Thermogravimetric Analysis (TGA)

TGA was performed using a TA Instruments Q50 thermogravimetric analyzer. The DMSO-swollen gels and the hydrogel samples were dried at room temperature, the former in a fume hood, for 48–72 h before analysis. The sample gas was air (flow rate = 60 mL/min) and the balance gas was N_2_ (flow rate = 40 mL/min). Samples were heated from room temperature to 1000 °C at a rate of 10 °C/min on a platinum pan.

### Calculating Zr Content from TGA Profiles

The Zr wt % in each sample was calculated from the final wt % of each sample at 1000 °C, as determined by TGA (Figures S22–S25 and Table S2). Since TGA was run in air, we assumed the inorganic fraction to be ZrO_2,_^51^ and calculated the wt % Zr in each sample by multiplying the final wt % (at 1000 °C) by the molecular weight fraction of Zr (91 g/mol) to ZrO_2_ (123 g/mol) (Table S2). The Zr wt % of each sample was calculated before and after solvent exchange, to track the loss of Zr throughout the process. As UiO-66 powder did not form large enough particles in DMSO to be separated from the supernatant, we could not analyze it using TGA. So, we could only analyze the UiO-66 powder after the dialysis.

### ^1^H Nuclear Magnetic Resonance Spectroscopy

^1^H NMR spectroscopy was conducted on a 400 MHz Varian NMR spectrometer unless otherwise noted. To quantify the DMSO content in the composite gels before and after dialysis, we digested the 3 wt % PVA-UiO-66 composite gels in 1 M sodium deuteroxide in deuterium oxide (D_2_O) at 10 mg/mL before and after dialysis. We then spiked the samples with 10 μL of methanol and used ^1^H NMR spectroscopy to quantify the amount of DMSO, comparing the integration of the DMSO proton resonances to those of methanol to determine their wt % within the composite gels and hydrogels.

## Supporting Information Available

Additional characterization of composite gels (inversion tests, scanning electron microscopy images, and X-ray diffraction patterns), characterization of the solvent exchange process, and details of the loading and release experiments (PDF)

## References

[ref1] Peterson G. W.; Au K.; Tovar T. M.; Epps T. H. Multivariate Cubtc Metal-Organic Framework with Enhanced Selectivity, Stability, Compatibility, and Processability. Chem. Mater. 2019, 31 (20), 8459–8465. 10.1021/acs.chemmater.9b02756.

[ref2] Sharma P.; Goswami R.; Neogi S.; Shahi V. K. Devising Ultra-Robust Mixed-Matrix Membrane Separators Using Functionalized MOF-Poly(Phenylene Oxide) for High-Performance Vanadium Redox Flow Batteries. J. Mater. Chem. A Mater. 2022, 10 (20), 11150–11162. 10.1039/D1TA10715A.

[ref3] Wang M.; Huang H.; Ma X.; Huang C.; Peng X. Copper Metal-Organic Framework Embedded Carboxymethyl Chitosan-g-Glutathione/Polyacrylamide Hydrogels for Killing Bacteria and Promoting Wound Healing. Int. J. Biol. Macromol. 2021, 187 (May), 699–709. 10.1016/j.ijbiomac.2021.07.139. 34331983

[ref4] Nie W.; Huang Y.; Wang Y.; Kengla C.; Scott Copus J.; Sun J.; Shao Z.; Dai X.; Shen Y. Temperature Sensitive PolyMOF Hydrogel Formed by in Situ Open-Ring Polymerization for Infected Chronic Wound Treatment. Chemical Engineering Journal 2022, 446 (P2), 136948 10.1016/j.cej.2022.136948.

[ref5] Gwon K.; Han I.; Lee S.; Kim Y.; Lee D. N. Novel Metal-Organic Framework-Based Photocrosslinked Hydrogel System for Efficient Antibacterial Applications. ACS Appl. Mater. Interfaces 2020, 12 (18), 20234–20242. 10.1021/acsami.0c03187. 32285658

[ref6] Javanbakht S.; Nabi M.; Shadi M.; Amini M. M.; Shaabani A. Carboxymethyl Cellulose/Tetracycline@UiO-66 Nanocomposite Hydrogel Films as a Potential Antibacterial Wound Dressing. Int. J. Biol. Macromol. 2021, 188 (May), 811–819. 10.1016/j.ijbiomac.2021.08.061. 34390748

[ref7] Denny M. S.; Cohen S. M. In Situ Modification of Metal-Organic Frameworks in Mixed-Matrix Membranes. Angewandte Chemie - International Edition 2015, 54 (31), 9029–9032. 10.1002/anie.201504077. 26073065

[ref8] Zhao R.; Ma T.; Zhao S.; Rong H.; Tian Y.; Zhu G. Uniform and Stable Immobilization of Metal-Organic Frameworks into Chitosan Matrix for Enhanced Tetracycline Removal from Water. Chem. Eng. J. 2020, 382, 122893 10.1016/j.cej.2019.122893.

[ref9] Mahmoud M. E.; Mohamed A. K. Novel Derived Pectin Hydrogel from Mandarin Peel Based Metal-Organic Frameworks Composite for Enhanced Cr(VI) and Pb(II) Ions Removal. Int. J. Biol. Macromol. 2020, 164, 920–931. 10.1016/j.ijbiomac.2020.07.090. 32673717

[ref10] Allegretto J. A.; Giussi J. M.; Moya S. E.; Azzaroni O.; Rafti M. Synthesis and Characterization of Thermoresponsive ZIF-8@PNIPAm-: Co-MAA Microgel Composites with Enhanced Performance as an Adsorption/Release Platform. RSC Adv. 2020, 10 (5), 2453–2461. 10.1039/C9RA09729E. 35496105 PMC9048415

[ref11] Lim J.; Lee E. J.; Choi J. S.; Jeong N. C. Diffusion Control in the in Situ Synthesis of Iconic Metal-Organic Frameworks within an Ionic Polymer Matrix. ACS Appl. Mater. Interfaces 2018, 10 (4), 3793–3800. 10.1021/acsami.7b17662. 29297676

[ref12] Klein S. E.; Sosa J. D.; Castonguay A. C.; Flores W. I.; Zarzar L. D.; Liu Y. Green Synthesis of Zr-Based Metal-Organic Framework Hydrogel Composites and Their Enhanced Adsorptive Properties. Inorg. Chem. Front 2020, 7 (24), 4813–4821. 10.1039/D0QI00840K. 33520236 PMC7839982

[ref13] Zhu H.; Zhang Q.; Zhu S. Alginate Hydrogel: A Shapeable and Versatile Platform for in Situ Preparation of Metal-Organic Framework-Polymer Composites. ACS Appl. Mater. Interfaces 2016, 8 (27), 17395–17401. 10.1021/acsami.6b04505. 27315047

[ref14] Pastore V. J.; Cook T. R.; Rzayev J. Polymer-MOF Hybrid Composites with High Porosity and Stability through Surface-Selective Ligand Exchange. Chem. Mater. 2018, 30 (23), 8639–8649. 10.1021/acs.chemmater.8b03881.

[ref15] Hess S. C.; Grass R. N.; Stark W. J. MOF Channels within Porous Polymer Film: Flexible, Self-Supporting ZIF-8 Poly(Ether Sulfone) Composite Membrane. Chem. Mater. 2016, 28 (21), 7638–7644. 10.1021/acs.chemmater.6b02499.

[ref16] Palomba J. M.; Wirth D. M.; Kim J. Y.; Kalaj M.; Clarke E. M.; Peterson G. W.; Pokorski J. K.; Cohen S. M. Strong, Ductile MOF-Poly(Urethane Urea) Composites. Chem. Mater. 2021, 33 (9), 3164–3171. 10.1021/acs.chemmater.0c04874.

[ref17] Unnikrishnan V.; Zabihi O.; Ahmadi M.; Li Q.; Blanchard P.; Kiziltas A.; Naebe M. Metal-Organic Framework Structure-Property Relationships for High-Performance Multifunctional Polymer Nanocomposite Applications. J. Mater. Chem. A Mater. 2021, 9 (8), 4348–4378. 10.1039/D0TA11255K.

[ref18] Lawson S.; Alwakwak A. A.; Rownaghi A. A.; Rezaei F. Gel-Print-Grow: A New Way of 3D Printing Metal-Organic Frameworks. ACS Appl. Mater. Interfaces 2020, 12 (50), 56108–56117. 10.1021/acsami.0c18720. 33274935

[ref19] Garai A.; Shepherd W.; Huo J.; Bradshaw D. Biomineral-Inspired Growth of Metal-Organic Frameworks in Gelatin Hydrogel Matrices. J. Mater. Chem. B 2013, 1 (30), 3678–3684. 10.1039/c3tb20814a. 32261265

[ref20] Zhuang Y.; Kong Y.; Wang X.; Shi B. Novel One Step Preparation of a 3D Alginate Based MOF Hydrogel for Water Treatment. New J. Chem. 2019, 43 (19), 7202–7208. 10.1039/C8NJ06031B.

[ref21] Wang Y.; Peng H.; Wang H.; Zhang M.; Zhao W.; Zhang Y. In-Situ Synthesis of MOF Nanoparticles in Double-Network Hydrogels for Stretchable Adsorption Device. Chemical Engineering Journal 2022, 450 (P3), 138216 10.1016/j.cej.2022.138216.

[ref22] Tang L.; Gong L.; Xu Y.; Wu S.; Wang W.; Zheng B.; Tang Y.; Zhang D.; Tang J.; Zheng J. Mechanically Strong Metal–Organic Framework Nanoparticle-Based Double Network Hydrogels for Fluorescence Imaging. ACS Appl. Nano Mater. 2022, 5 (1), 1348–1355. 10.1021/acsanm.1c03034.

[ref23] Destefano M. R.; Islamoglu T.; Garibay S. J.; Hupp J. T.; Farha O. K. Room-Temperature Synthesis of UiO-66 and Thermal Modulation of Densities of Defect Sites. Chem. Mater. 2017, 29, 1357–1361. 10.1021/acs.chemmater.6b05115.

[ref24] Huelsenbeck L.; Luo H.; Verma P.; Dane J.; Ho R.; Beyer E.; Hall H.; Geise G. M.; Giri G. Generalized Approach for Rapid Aqueous MOF Synthesis by Controlling Solution PH. Cryst. Growth Des 2020, 20 (10), 6787–6795. 10.1021/acs.cgd.0c00895.

[ref25] Somjit V.; Thinsoongnoen P.; Waiprasoet S.; Pila T.; Pattanasattayavong P.; Horike S.; Kongpatpanich K. Processable UiO-66 Metal-Organic Framework Fluid Gel and Electrical Conductivity of Its Nanofilm with Sub-100 Nm Thickness. ACS Appl. Mater. Interfaces 2021, 13 (26), 30844–30852. 10.1021/acsami.1c07262. 34165275

[ref26] Patterson A. L. The Scherrer Formula for X-Ray Particle Size Determination. Phys. Rev. 1939, 56 (10), 978–982. 10.1103/PhysRev.56.978.

[ref27] Dighe A. V.; Huelsenbeck L.; Bhawnani R. R.; Verma P.; Stone K. H.; Singh M. R.; Giri G. Autocatalysis and Oriented Attachment Direct the Synthesis of a Metal-Organic Framework. JACS Au 2022, 2 (2), 453–462. 10.1021/jacsau.1c00494. 35252994 PMC8889615

[ref28] MacRae C. F.; Sovago I.; Cottrell S. J.; Galek P. T. A.; McCabe P.; Pidcock E.; Platings M.; Shields G. P.; Stevens J. S.; Towler M.; Wood P. A. Mercury 4.0: From Visualization to Analysis, Design and Prediction. J. Appl. Crystallogr. 2020, 53, 226–235. 10.1107/S1600576719014092. 32047413 PMC6998782

[ref29] Deblock L.; Goossens E.; Pokratath R.; De Buysser K.; De Roo J. Mapping out the Aqueous Surface Chemistry of Metal Oxide Nanocrystals: Carboxylate, Phosphonate, and Catecholate Ligands. JACS Au 2022, 2 (3), 711–722. 10.1021/jacsau.1c00565. 35373200 PMC8969999

[ref30] Verma P. K.; Huelsenbeck L.; Nichols A. W.; Islamoglu T.; Heinrich H.; Machan C. W.; Giri G. Controlling Polymorphism and Orientation of NU-901/NU-1000 Metal-Organic Framework Thin Films. Chem. Mater. 2020, 32 (24), 10556–10565. 10.1021/acs.chemmater.0c03539.

[ref31] Chavan S.; Vitillo J. G.; Gianolio D.; Zavorotynska O.; Civalleri B.; Jakobsen S.; Nilsen M. H.; Valenzano L.; Lamberti C.; Lillerud K. P.; Bordiga S. H 2 Storage in Isostructural UiO-67 and UiO-66 MOFs. Phys. Chem. Chem. Phys. 2012, 14 (5), 1614–1626. 10.1039/C1CP23434J. 22187720

[ref32] Morris W.; Volosskiy B.; Demir S.; Gándara F.; McGrier P. L.; Furukawa H.; Cascio D.; Stoddart J. F.; Yaghi O. M. Synthesis, Structure, and Metalation of Two New Highly Porous Zirconium Metal-Organic Frameworks. Inorg. Chem. 2012, 51 (12), 6443–6445. 10.1021/ic300825s. 22676251

[ref33] Islamoglu T.; Otake K. I.; Li P.; Buru C. T.; Peters A. W.; Akpinar I.; Garibay S. J.; Farha O. K. Revisiting the Structural Homogeneity of NU-1000, a Zr-Based Metal–Organic Framework. CrystEngComm 2018, 20 (39), 5913 10.1039/C8CE00455B.

[ref34] Song X.; Yang P.; Wu D.; Zhao P.; Zhao X.; Yang L.; Zhou Y. Facile Synthesis of Metal-Organic Framework UiO-66 for Adsorptive Removal of Methylene Blue from Water. Chem. Phys. 2020, 531, 110655 10.1016/j.chemphys.2019.110655.

[ref35] Mohammadi A. A.; Alinejad A.; Kamarehie B.; Javan S.; Ghaderpoury A.; Ahmadpour M.; Ghaderpoori M. Metal-Organic Framework Uio-66 for Adsorption of Methylene Blue Dye from Aqueous Solutions. Int. J. Environ. Sci. Technol. 2017, 14 (9), 1959–1968. 10.1007/s13762-017-1289-z.

[ref36] Muttenthaler M.; King G. F.; Adams D. J.; Alewood P. F. Trends in Peptide Drug Discovery. Nat. Rev. Drug Discovery 2021, 20, 309–325. 10.1038/s41573-020-00135-8. 33536635

[ref37] Fosgerau K.; Hoffmann T. Peptide Therapeutics: Current Status and Future Directions. Drug Discov Today 2015, 20 (1), 122–128. 10.1016/j.drudis.2014.10.003. 25450771

[ref38] Lau J. L.; Dunn M. K. Therapeutic Peptides: Historical Perspectives, Current Development Trends, and Future Directions. Bioorg. Med. Chem. 2018, 26 (10), 2700–2707. 10.1016/j.bmc.2017.06.052. 28720325

[ref39] Di L. Strategic Approaches to Optimizing Peptide ADME Properties. AAPS Journal 2015, 17 (1), 134–143. 10.1208/s12248-014-9687-3. 25366889 PMC4287298

[ref40] Petty W. J.; Miller A. A.; McCoy T. P.; Gallagher P. E.; Tallant E. A.; Torti F. M. Phase I and Pharmacokinetic Study of Angiotensin-(1–7), an Endogenous Antiangiogenic Hormone. Clin. Cancer Res. 2009, 15 (23), 7398–7404. 10.1158/1078-0432.CCR-09-1957. 19920106 PMC3703919

[ref41] Jiang F.; Yang J.; Zhang Y.; Dong M.; Wang S.; Zhang Q.; Liu F. F.; Zhang K.; Zhang C. Angiotensin-Converting Enzyme 2 and Angiotensin 1–7: Novel Therapeutic Targets. Nat. Rev. Cardiol. 2014, 11, 413–426. 10.1038/nrcardio.2014.59. 24776703 PMC7097196

[ref42] Zhou Y.; Liu L.; Cao Y.; Yu S.; He C.; Chen X. A Nanocomposite Vehicle Based on Metal-Organic Framework Nanoparticle Incorporated Biodegradable Microspheres for Enhanced Oral Insulin Delivery. ACS Appl. Mater. Interfaces 2020, 12 (20), 22581–22592. 10.1021/acsami.0c04303. 32340452

[ref43] Wang T. C.; Vermeulen N. A.; Kim I. S.; Martinson A. B. F.; Stoddart J. F.; Hupp J. T.; Farha O. K. Scalable Synthesis and Post-Modification of a Mesoporous Metal-Organic Framework Called NU-1000. Nat. Protoc. 2016, 11 (1), 149–162. 10.1038/nprot.2016.001. 26678084

[ref44] Thompson A. P.; Aktulga H. M.; Berger R.; Bolintineanu D. S.; Brown W. M.; Crozier P. S.; in’t Veld P. J.; Kohlmeyer A.; Moore S. G.; Nguyen T. D.; Shan R.; Stevens M. J.; Tranchida J.; Trott C.; Plimpton S. J. LAMMPS - a Flexible Simulation Tool for Particle-Based Materials Modeling at the Atomic, Meso, and Continuum Scales. Comput. Phys. Commun. 2022, 271, 108171 10.1016/j.cpc.2021.108171.

[ref45] Boyd P. G.; Moosavi S. M.; Witman M.; Smit B. Force-Field Prediction of Materials Properties in Metal-Organic Frameworks. J. Phys. Chem. Lett. 2017, 8 (2), 357–363. 10.1021/acs.jpclett.6b02532. 28008758 PMC5253710

[ref46] Rappé A. K.; Casewit C. J.; Colwell K. S.; Goddard W. A.; Skiff W. M. UFF, a Full Periodic Table Force Field for Molecular Mechanics and Molecular Dynamics Simulations. J. Am. Chem. Soc. 1992, 114 (25), 10024–10035. 10.1021/ja00051a040.

[ref47] Darve E.; Rodríguez-Gómez D.; Pohorille A. Adaptive Biasing Force Method for Scalar and Vector Free Energy Calculations. J. Chem. Phys. 2008, 128 (14), 144120 10.1063/1.2829861. 18412436

[ref48] Sidky H.; Colón Y. J.; Helfferich J.; Sikora B. J.; Bezik C.; Chu W.; Giberti F.; Guo A. Z.; Jiang X.; Lequieu J.; Li J.; Moller J.; Quevillon M. J.; Rahimi M.; Ramezani-Dakhel H.; Rathee V. S.; Reid D. R.; Sevgen E.; Thapar V.; Webb M. A.; Whitmer J. K.; De Pablo J. J. SSAGES: Software Suite for Advanced General Ensemble Simulations. J. Chem. Phys. 2018, 148 (4), 044104 10.1063/1.5008853. 29390830

[ref49] Vanden-Eijnden E.; Venturoli M. Revisiting the Finite Temperature String Method for the Calculation of Reaction Tubes and Free Energies. J. Chem. Phys. 2009, 130 (19), 194103 10.1063/1.3130083. 19466817

[ref50] Sarkisov L.; Bueno-Perez R.; Sutharson M.; Fairen-Jimenez D. Materials Informatics with PoreBlazer v4.0 and the CSD MOF Database. Chem. Mater. 2020, 32 (23), 9849–9867. 10.1021/acs.chemmater.0c03575.

[ref51] Athar M.; Rzepka P.; Thoeny D.; Ranocchiari M.; Anton Van Bokhoven J. Thermal Degradation of Defective High-Surface-Area UiO-66 in Different Gaseous Environments. *RSC*. Advances 2021, 11 (61), 38849–38855. 10.1039/D1RA05411B. PMC9044256 35493258

[ref52] Knebel A.; Sundermann L.; Mohmeyer A.; Strauß I.; Friebe S.; Behrens P.; Caro J. Azobenzene Guest Molecules as Light-Switchable CO_2_ Valves in an Ultrathin UiO-67 Membrane. Chem. Mater. 2017, 29 (7), 3111–3117. 10.1021/acs.chemmater.7b00147.

