# Supplementary material for: Expanding the Design Space of Polymer–Metal Organic Framework (MOF) Gels by Understanding Polymer–MOF Interactions
Source: Chem Mater. 2024 Jul 25;36(19):9356–69. doi: 10.1021/acs.chemmater.4c00112 (PMC11467831; doi:10.1021/acs.chemmater.4c00112)
Supplement: Supplementary file 1 — cm4c00112_si_001.pdf [file cm4c00112_si_001.pdf]

Supplemental Information

Expanding the design space of polymer-metal organic framework (MOF) gels by understanding polymer-MOF interactions

Prince Verma[+,1], Mark S. Bannon[+,1], Mara K. Kuenen[1], Sanoj Raj[2], Ankit Dhakal[1], Kevin Stone[3], Asa W. Nichols[4], Charles W. Machan[4], Yamil J. Colón[2], Rachel A. Letteri[*,1], Gaurav Giri[*,1]

[1]Department of Chemical Engineering, University of Virginia, Charlottesville, Virginia 22903, United States

[2]Department of Chemical and Biomolecular Engineering, University of Notre Dame, Notre Dame, Indiana 46556, United States

[3]Stanford Synchrotron Radiation Lightsource, SLAC National Accelerator Laboratory, Menlo Park, California 94025, United States

[4]Department of Chemistry, University of Virginia, Charlottesville, Virginia 22904, United States

[+]Equal Contributors

***Corresponding Authors**
rl2gm@virginia.edu (Rachel A. Letteri)
gg3qd@virginia.edu (Gaurav Giri)

Table of Contents

Section S1. Characterization of UiO-66-based composite gels and controls

Section S1a. Characterization of UiO-66

Figure S1. Formation of UiO-66 particles in DMSO

Figure S2. High-magnification scanning electron microscopy (SEM) images of UiO-66 synthesized in DMSO.

Section S1b. Polymer-UiO-66 composite gel formation with different linker:metal ratios

Figure S3. Inversion test of PVA-UiO-66 composites synthesized at a linker:metal ratio of 1:1 and 2:1

Section S1c. Characterization of PAA-based composite gels

Figure S4. High-magnification SEM image of PAA-UiO-66 gel

Section S1d. Inversion test of polymer-UiO-66 physical mixtures

Figure S5. Inversion test of physical mixtures of PAA and UiO-66, PAAA and UiO-66, PVA and UiO-66, and PEG and UiO-66.

Section S1e. Characterization of PAAA-based composite gels

Figure S6. High-magnification SEM image of PAAA-UiO-66 composite gel

Section S1f. Characterization of PVA-based composite gels

Figure S7. High-magnification SEM image of PVA-UiO-66 composite gel

Figure S8. Inversion tests of PVA (146000-186000 g/mol) - UiO-66 formulations as a function of polymer concentration

Figure S9. Inversion tests of PVA (31000-50000 g/mol) – UiO-66 formulations

Figure S10. Inversion test of PVA (9000-10000 g/mol) - UiO-66 formulations

Figure S11. Phase diagram of PVA-UiO-66 formulations with different concentrations (wt%) and molecular weight of PVA

Section S1g. Characterization of PEG-based composite gels

Figure S12. High-magnification SEM image of PEG-UiO-66 composite gel

Figure S13. Composite gel stability at room temperature and after heating to 40 ºC

Section S2. Characterization of PVA-based composite gels prepared with a variety of Zr-based MOFs

Figure S14. ^{1}H NMR of 4,4',4'',4'''-(pyrene-1,3,6,8-tetrayl)tetrabenzoic acid (H_4TBAPy)

Figure S15. High-magnification SEM image of PVA-NU-901 composite gel

Figure S16. High-magnification SEM image of PVA-UiO-67 composite gel

Figure S17. High-magnification SEM image of PVA-MOF-525 composite gel

Figure S18. PVA-NU-901, PVA-UiO-67, and PVA-MOF-525 composite gel stability at room temperature after heating to 40 ºC for 24 h

Section S3. Composite gel solvent exchange

Figure S19. Gelation behavior of 2 wt% PVA-UiO-66 composite synthesized in DI water.

Figure S20. Quantification of dimethylsulfoxide (DMSO) removal during composite gel solvent switch

Table S1. Quantification of DMSO in polymer-MOF composite hydrogels
Figure S21. GIXD patterns of 3 wt% PVA-MOF-525 and PVA-UiO-66 composite gels in DMSO and hydrogels in water.
Figure S22. Zr content before and after solvent exchange of PVA-UiO-66 and PVA-Zr-oxo gels from DMSO (yellow) into water (blue) by dialysis, as determined by TGA
Table S2. ZrO_2 and Zr wt% of PVA-UiO-66 composite gels, PVA-Zr-oxo gels, and UiO-66 powder before and after solvent exchange.
Figure S23. TGA profiles of 3 wt% PVA-UiO-66 composite gels before and after dialysis
Figure S24. TGA profiles of 3 wt% PVA-Zr-oxo gels before and after dialysis
Figure S25. TGA profiles of UiO-66 powder synthesized in DMSO
Section S4. MB sorption into and release from PVA-MOF composite gels
Figure S26. Calibration curves for MB and Ang 1-7 encapsulation and release experiments
Figure S27. MB sorption profiles of PVA-UiO-66 composite hydrogels and PVA-Zr-oxo hydrogels over 7 d, normalized by the dry weight of the carrier
Figure S28. Encapsulation efficiency, or % of MB in solution sorbed, of tested carriers after 7 d
Figure S29. MB sorptive capacity of various PVA-MOF composite hydrogels
Table S3. MB sorption into PVA-UiO-66 composite hydrogels, PVA-Zr-oxo hydrogels, and UiO-66 powder
Table S4. Reported values and conditions for UiO-66 MB sorptive capacities in literature.
Section S5. Ang 1-7 sorption into and release from PVA-MOF-525 composite gels
Figure S30. Characterization of Ang 1-7 peptide
Figure S31. Reverse phase high performance liquid chromatography depicting Ang 1-7 encapsulation into UiO-67 and MOF-525
Figure S32. Encapsulation efficiency, or % of Ang 1-7 in solution sorbed, of PVA-MOF 525 composite hydrogels, PVA-Zr-oxo hydrogels, and MOF-525 powder
Figure S33. Sorptive capacity and release behavior of PVA-MOF-525 composite hydrogels, PVA-Zr-oxo hydrogels, and MOF-525 powder for the therapeutic peptide Ang 1-7.
Table S5. Ang 1-7 sorption into PVA-MOF-525 composite hydrogels, PVA-Zr-oxo hydrogels, and MOF-525 powder
Figure S34. % of Ang 1-7 sorbed into PVA-MOF-525 composite hydrogels PVA-Zr-oxo hydrogels, and MOF-525 powder released into ultrapure water.
Table S6. % of Ang 1-7 sorbed into PVA-MOF-525 composite hydrogels, PVA-Zr-oxo hydrogels, and MOF-525 powder released into ultrapure water.

Section S1: Characterization of UiO-66 composite gels and controls

Section S1a: Characterization of UiO-66

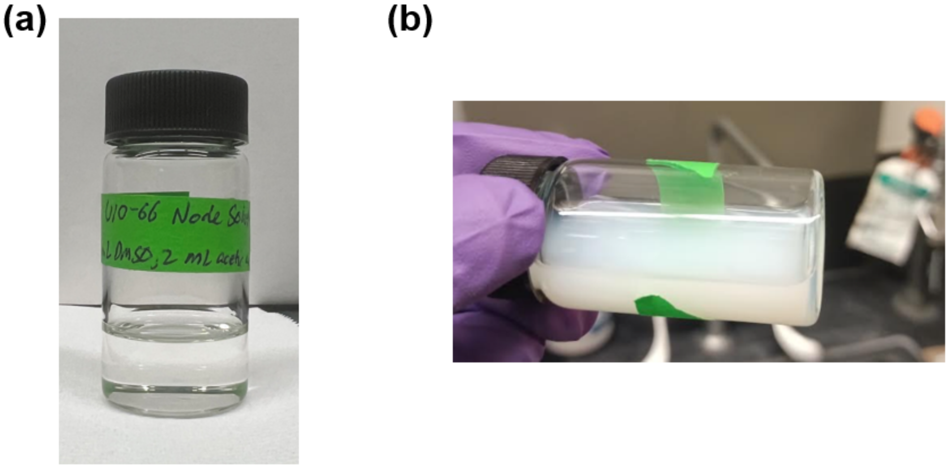

Figure S1. Formation of UiO-66 particles in DMSO. **(a)** Zr-oxo cluster solution in DMSO, which is transparent. **(b)** Formation of UiO-66 particles in DMSO as evidenced by the solution turning white upon the addition of H_2BDC and the GIXD pattern closely resembling the simulated pattern for UiO-66 (**Figure 1a** in manuscript).

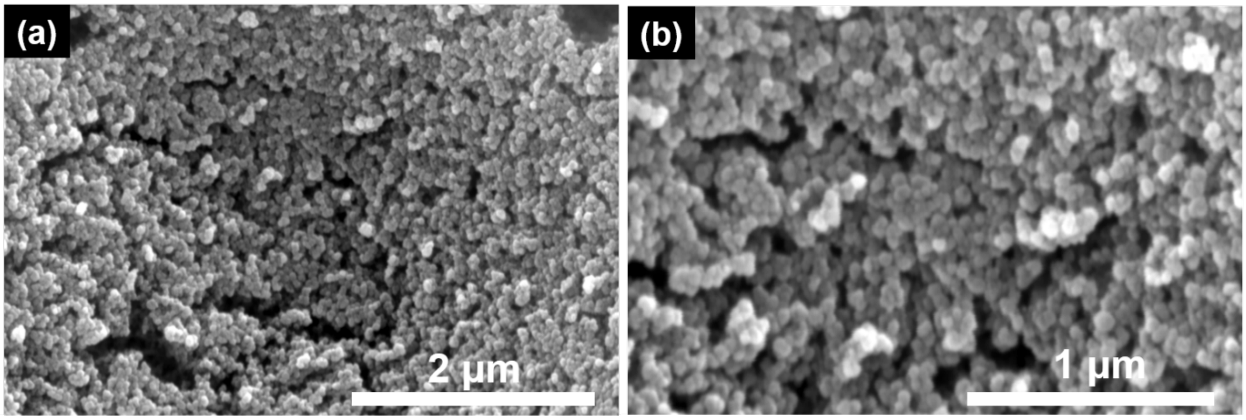

Figure S2. High-magnification scanning electron microscopy (SEM) images of UiO-66 synthesized in DMSO show the formation of spherical particles. Bars in bottom right corner represent **a)** 2 µm and **b)** 1 µm.

Section S1b: Polymer-UiO-66 composite gel formation with different linker: metal ratios

Since UiO-66 particles were synthesized at linker: metal ratio of 1:1, we attempted to form the polymer-UiO-66 composites at the same ratio. For this, we chose PVA as a polymer. To make the composite gels at a 1:1 linker: metal ratio, we added a solution of Zr-oxo clusters (70 mM in 5 mL of DMSO) to the PVA-H_2BDC solution (which contained 72 mM H_2BDC in 5 mL of DMSO) at room temperature for 24 h. No gelation occurred after 24 h, as suggested by the inversion test (**Figure S3a**). On the contrary, doubling the H_2BDC concentration in the PVA-H_2BDC solution 144 mM H_2BDC in 5 mL of DMSO) and adding the Zr-oxo cluster solution at room temperature resulted in gelation after 24 h (**Figure S3b**). Therefore, we synthesized all the polymer-UiO-66 composite gels (PAA-UiO-66, PAAA-UiO-66, PVA-UiO-66, and PEG-UiO-66) at a linker: metal ratio of 2:1.

We avoided lowering the linker:metal ratio, like 1:2 or 1:4 to form composite gels. We suspect that lowering the linker concentration slows down the UiO-66 crystal formation, which might result in crystals with less defects/open metal sites.[14] Since we suspect that defects are required for gel formation, lowering linker concentration might not produce gel network. Another way to lower the linker:metal ratio is to increase the Zr-oxo cluster concentration. However, we are already using a very high concentration of Zr-oxo clusters in DMSO. Further increasing the Zr-oxo concentration might not be possible due to the solubility limitations of Zr propoxide in DMSO. Additionally, adding more Zr propoxide might change the equilibrium of Zr_6 species and may produce other unfavorable Zr species not suitable for UiO-66 formation.[15] Zr-oxo cluster formation is a complex process and it requires further study.

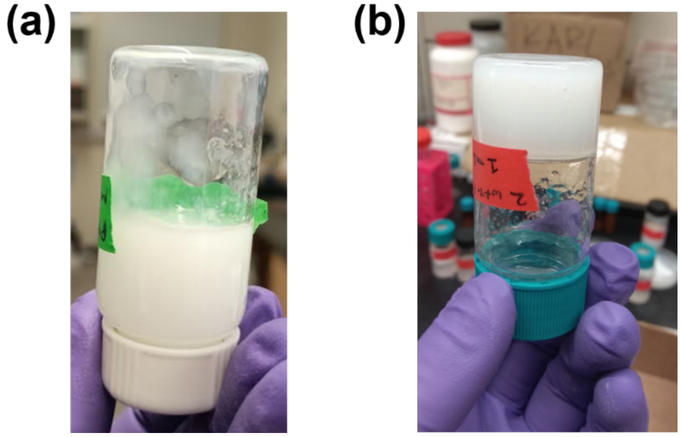

Figure S3. Inversion test of PVA-UiO-66 composites synthesized at a linker: metal ratio of (a) 1:1 and (b) 2:1. The gelation occurs at linker: metal ratio of 2:1. The PVA wt% is 2 for both cases. The images were taken one min after inverting the vials.

Section S1c: Characterization of PAA-based composite gels

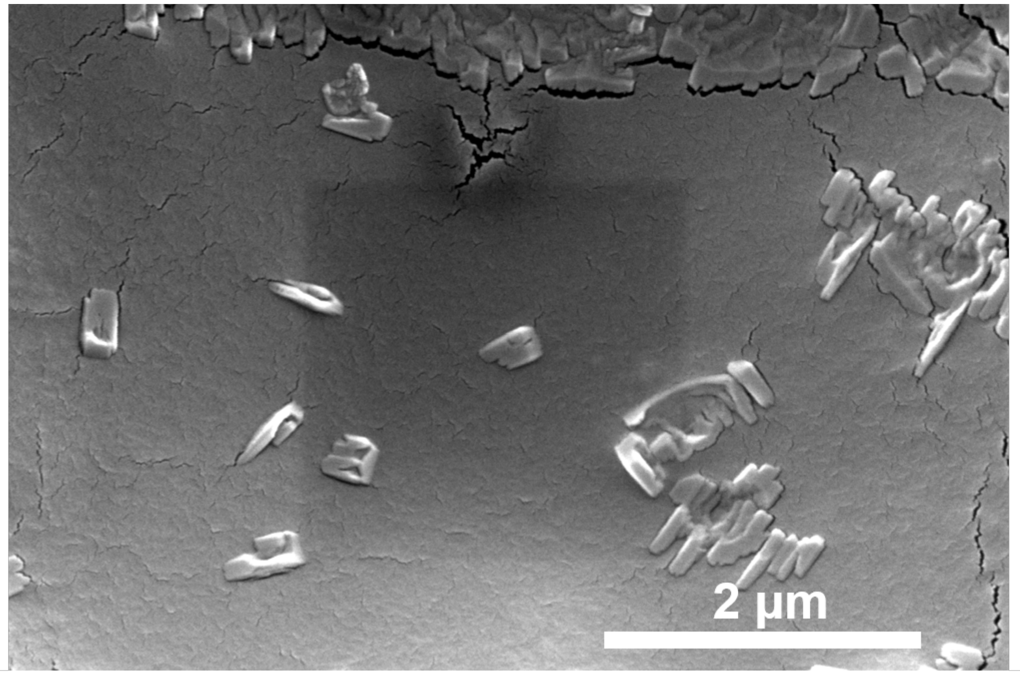

Figure S4. High-magnification SEM image of PAA-UiO-66 gel shows no spherical particles such as those observed in Figure S2 for UiO-66 particles synthesized in DMSO in the absence of PAA. The absence of spherical particles in the PAA-UiO-66 gels suggests no UiO-66 formation in the presence of PAA.

Section S1d: Inversion test of polymer-UiO-66 physical mixtures

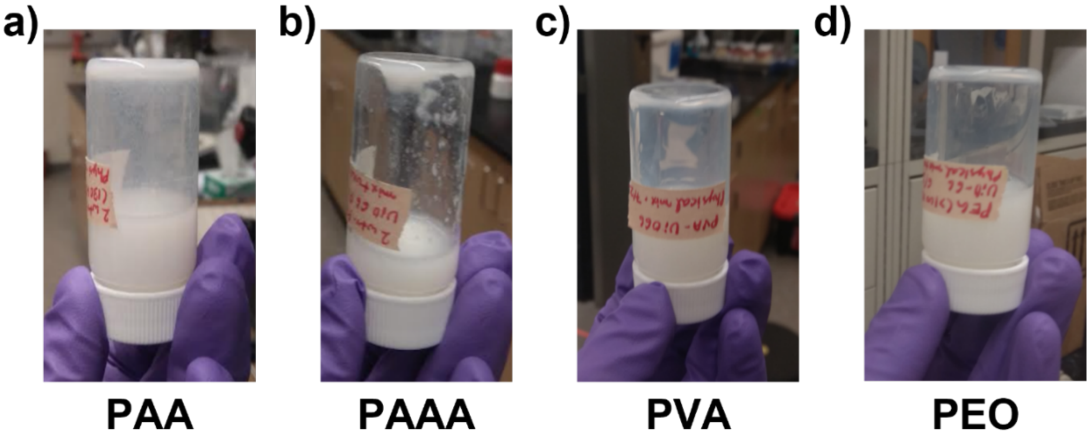

Figure S5. Inversion tests of physical mixtures of a) PAA and UiO-66, b) PAAA and UiO-66, c) PVA and UiO-66, and d) PEG and UiO-66 showing the absence of gelation when pre-formed MOFs are mixed with polymers. Photographs were taken one minute after inverting the vials.

Section S1e: Characterization of PAAA-based composite gels

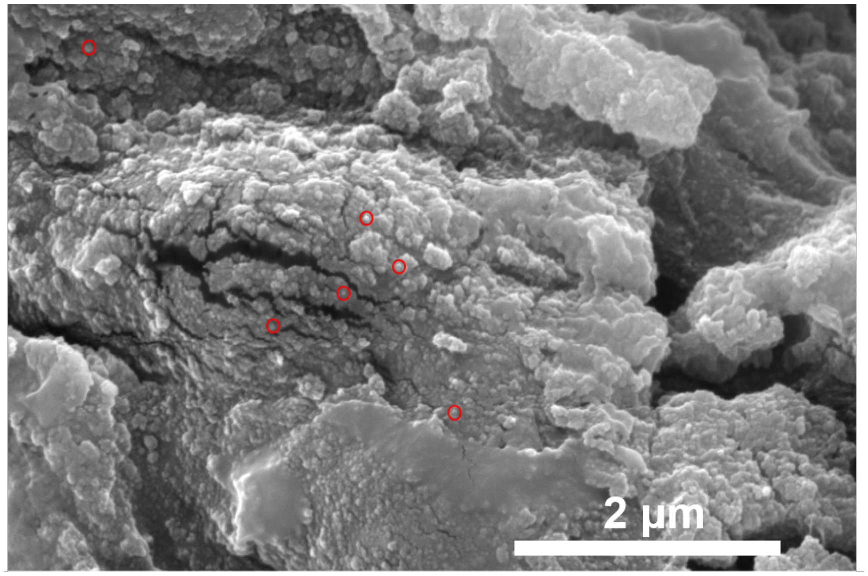

Figure S6. High-magnification SEM image of PAAA-UiO-66 composite gel showing the presence of spherical particles, suggesting the formation of UiO-66 in the presence of PAAA. Red circles show the UiO-66 particles within the composite gel.

Section S1f: Characterization of PVA-based composite gels

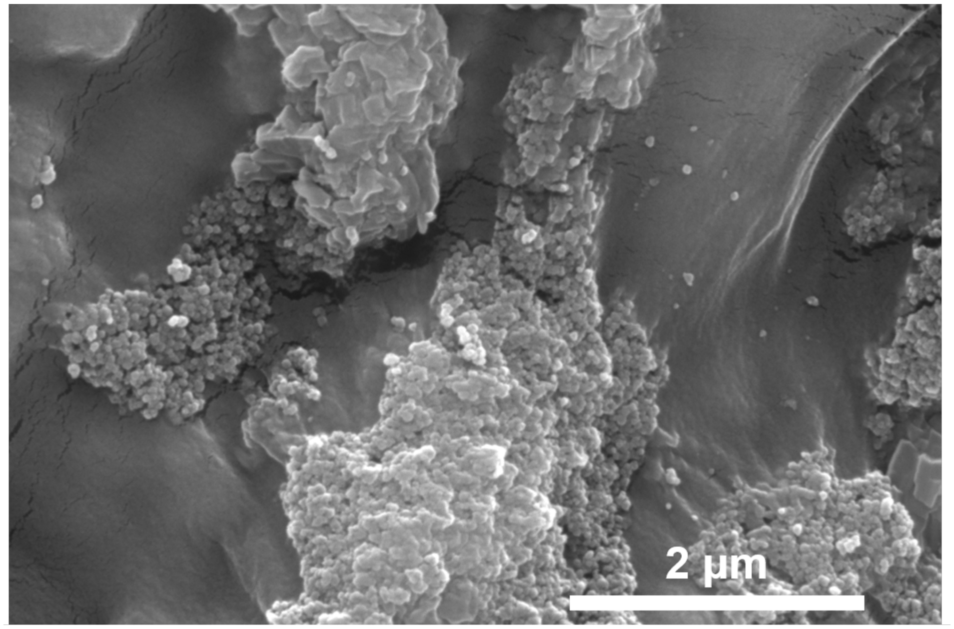

Figure S7. High-magnification SEM image of PVA-UiO-66 composite gel showing the presence of spherical particles, suggesting the formation of UiO-66 in the presence of PVA.

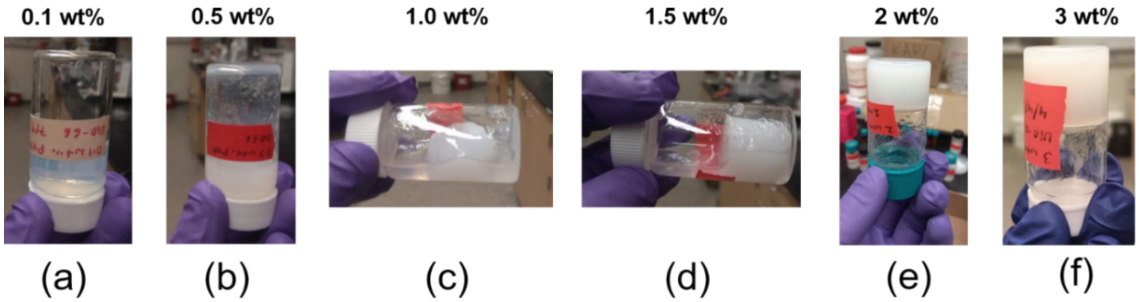

Figure S8. Inversion tests of PVA (146000-186000 g/mol) - UiO-66 formulations as a function of polymer concentration: (a) 0.1; (b) 0.5; (c) 1.0; (d) 1.5; (e) 2; and (f) 3 wt% PVA. The study shows that a critical PVA concentration (1.5-2 wt%) is required to induce gelation. Images were taken one min after inverting the vial.

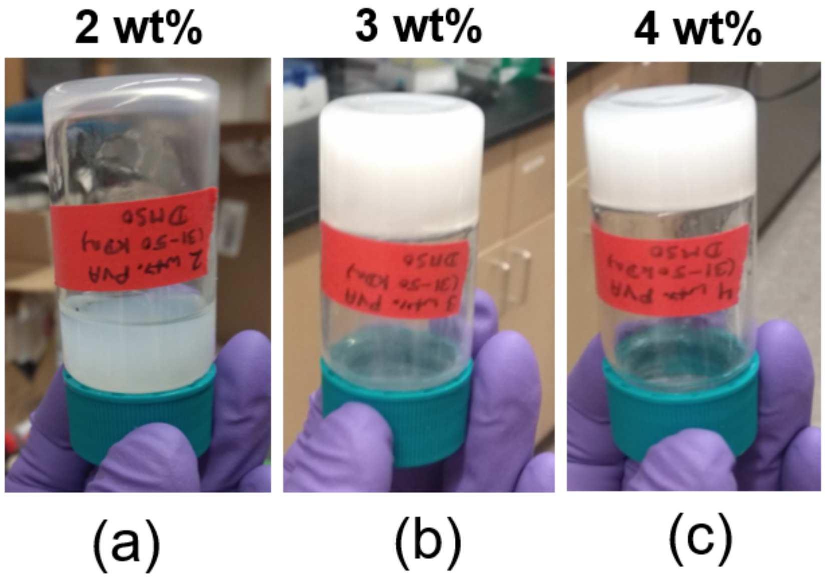

Figure S9. Inversion tests of PVA (31000-50000 g/mol) – UiO-66 formulations at (a) 2, (b) 3, and (c) 4 wt% PVA. For this lower molecular weight PVA, gelation requires at least 3 wt% PVA, slightly higher than the concentration needed for gelation using the higher molecular weight PVA (146000-186000 g/mol). Images were taken one min after inverting the vial.

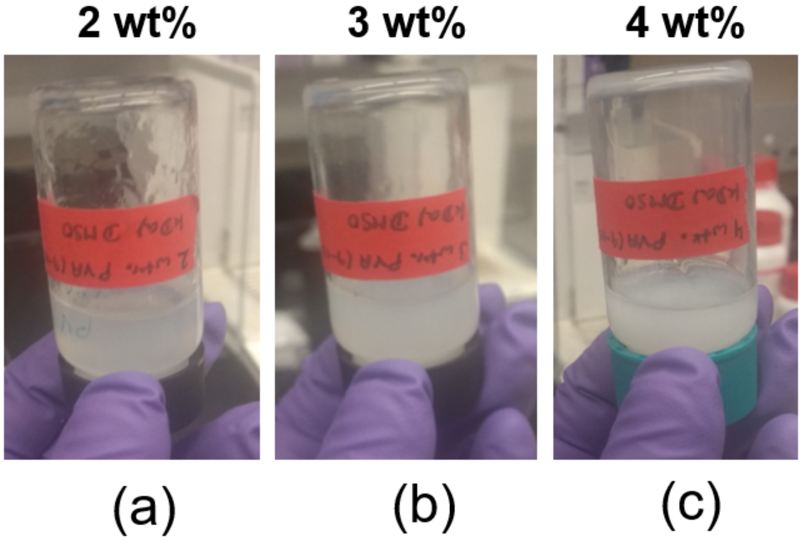

Figure S10. Inversion test of PVA (9000-10000 g/mol) - UiO-66 formulations at (a) 2, (b) 3, and (c) 4 wt% PVA. No gelation was observed, even at 4 wt% PVA using this low molecular weight polymer. Images were taken one min after inverting the vial.

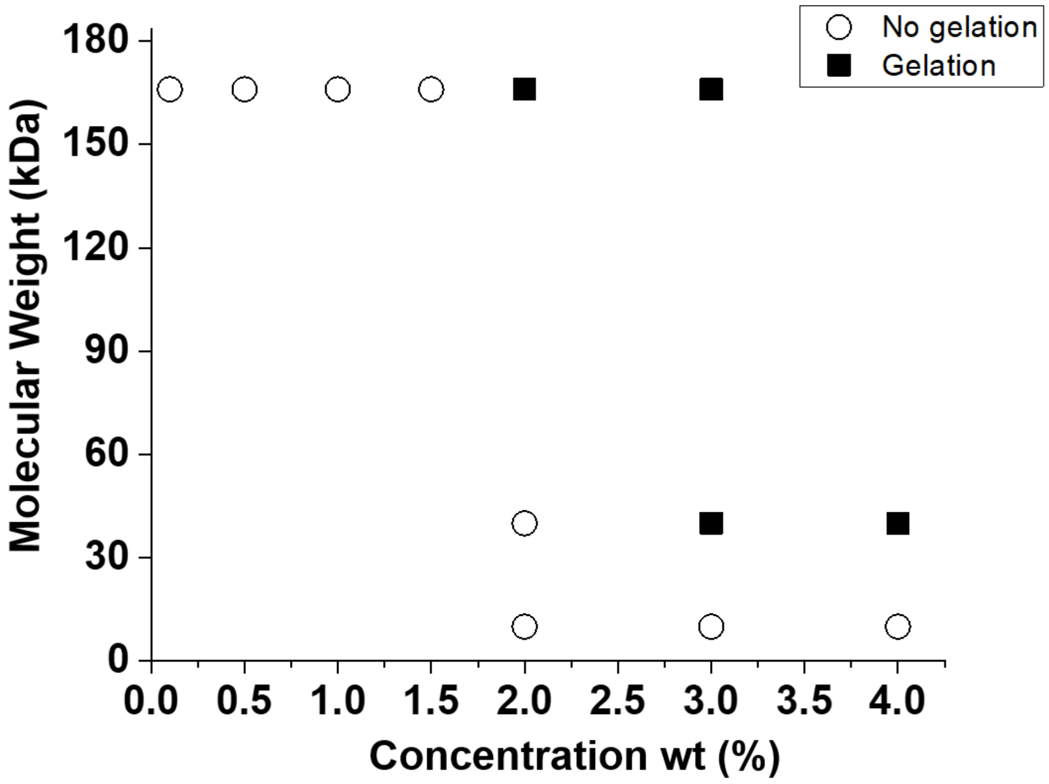

Figure S11. Phase diagram of PVA-UiO-66 formulations with different concentrations (wt%) and molecular weight of PVA demonstrating the conditions where no gelation and gelation occurred.

Section S1g: Characterization of PEG-based composite gels

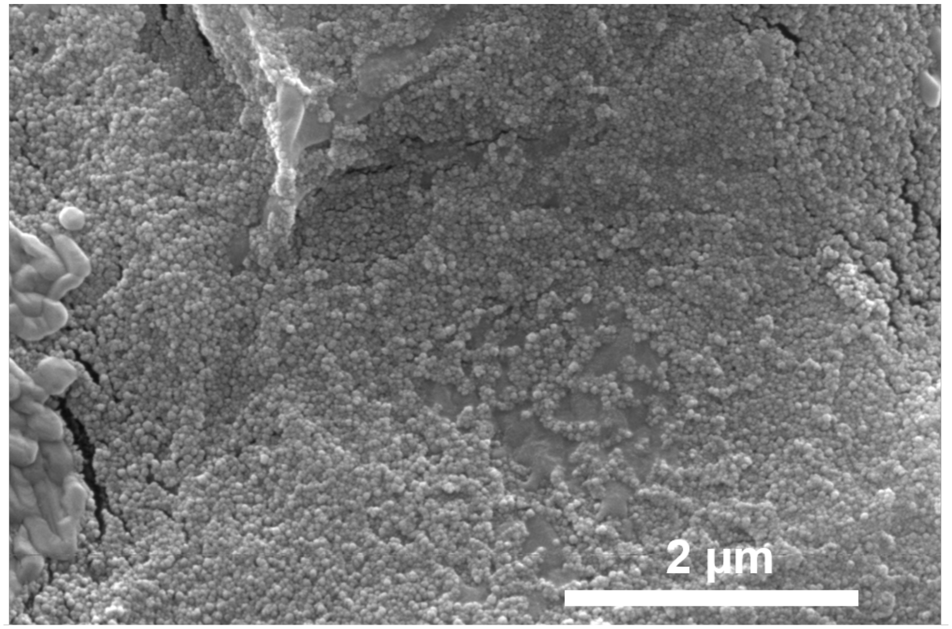

Figure S12. High-magnification SEM image of PEG-UiO-66 composite gel shows the presence of spherical particles, suggesting the formation of UiO-66 in the presence of PEG.

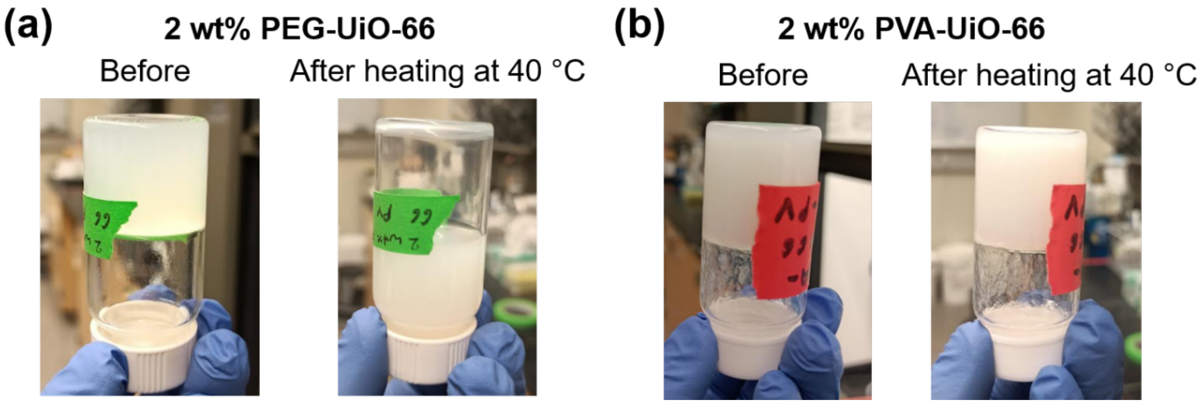

Figure S13. Composite gel stability at room temperature and after heating to 40 ºC. (a) PEG-UiO-66 composite gel collapses after heating at 40 °C for 24 h, while (b) PVA-UiO-66 composite gel remains intact. This experiment suggests that heating promotes the reptation of PEG chains out of UiO-66 pores and the PEG-UiO-66 composite collapses. Therefore, entrapment of PEG chains inside UiO-66 is responsible for gelation.

Section S2: Characterization of PVA-based composite gels prepared with a variety of Zr-based MOFs

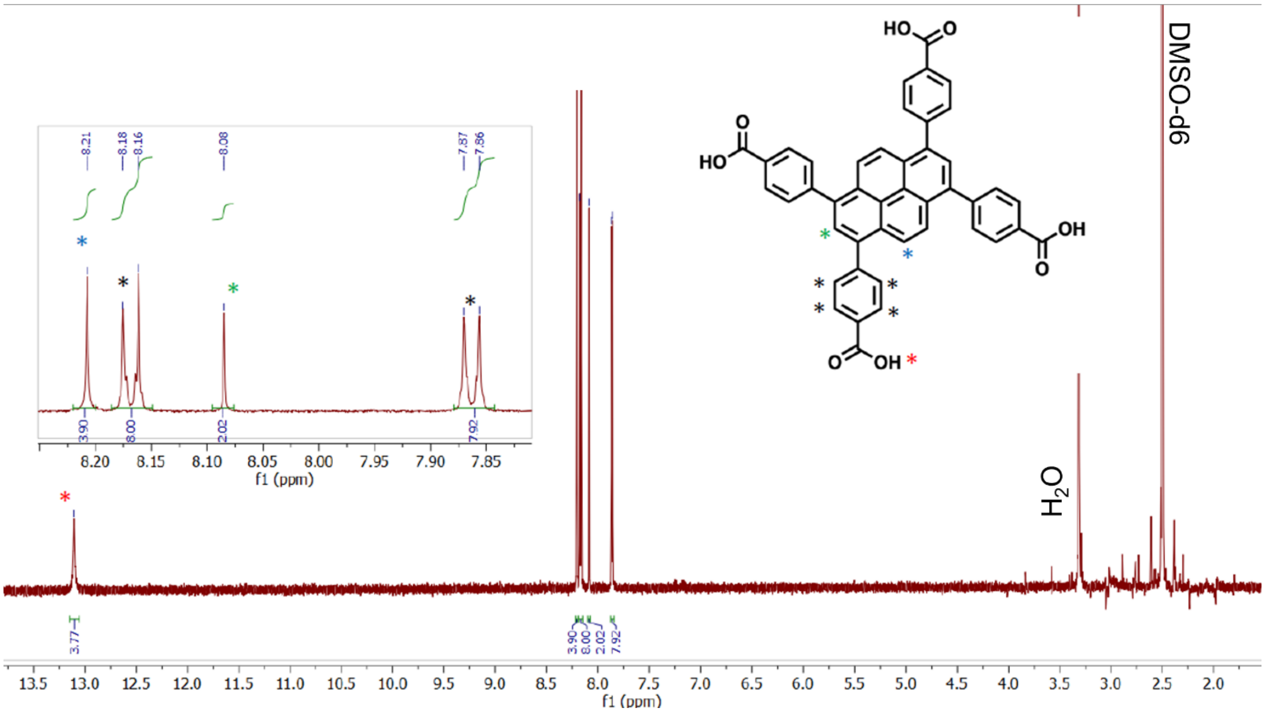

Figure S14. ^{1}H NMR (600 MHz, DMSO-d6) of 4,4',4'',4'''-(pyrene-1,3,6,8-tetrayl)tetrabenzoic acid (H_4TBAPy), the organic linker for NU-901.

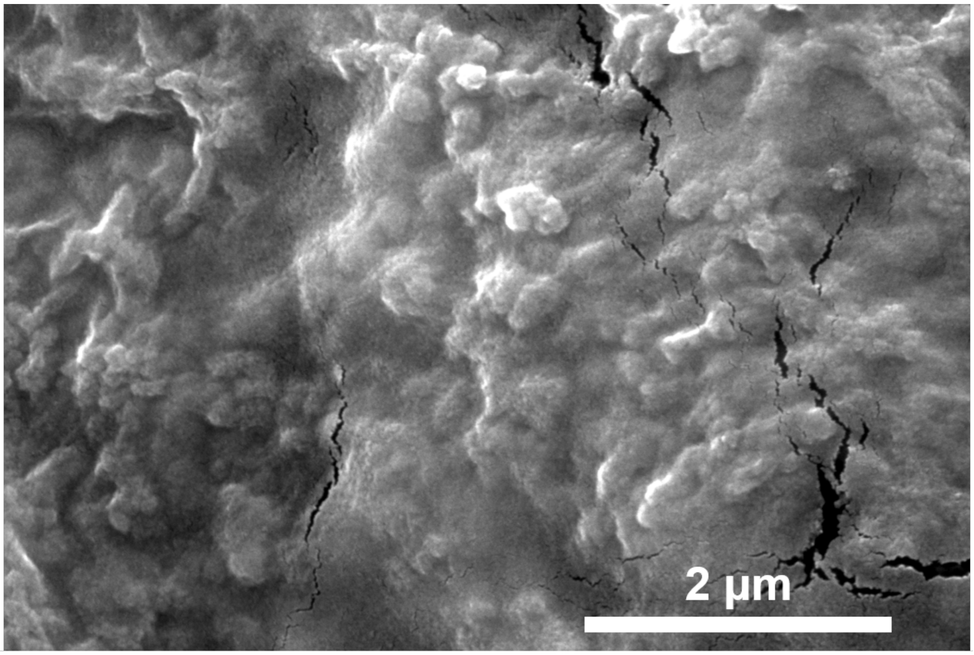

Figure S15. High-magnification SEM image of PVA-NU-901 composite gel showing the presence of spherical particles, suggesting the formation of NU-901 in the presence of PVA.

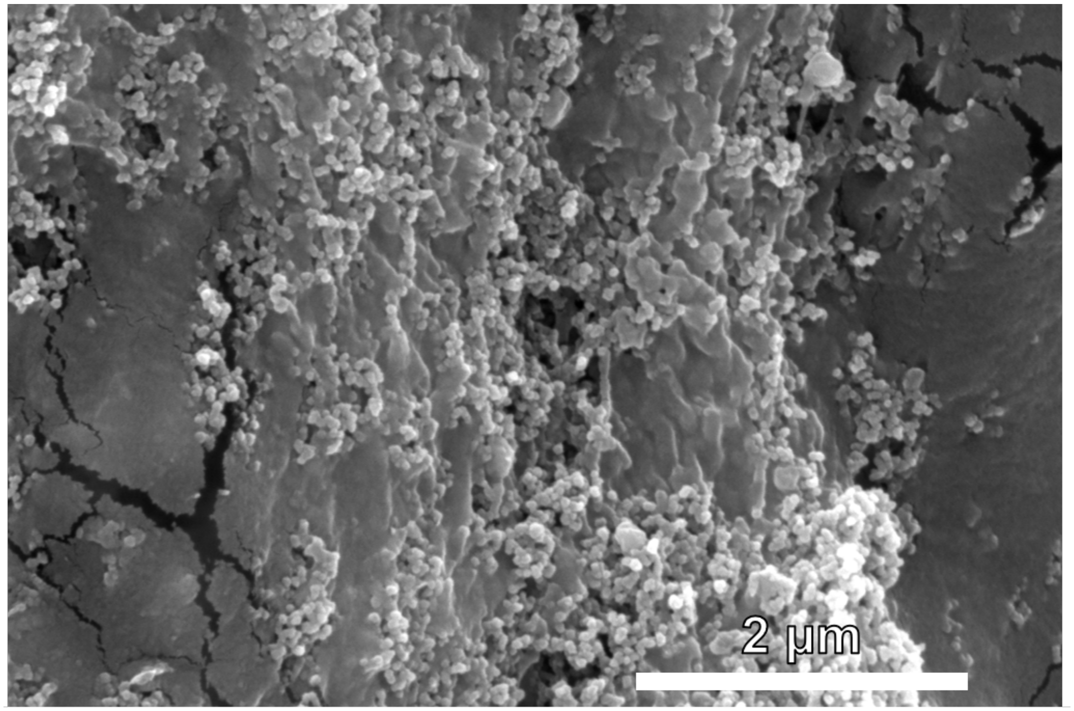

Figure S16. High-magnification SEM image of PVA-UiO-67 composite gel shows the presence of spherical particles, suggesting the formation of UiO-67 in the presence of PVA.

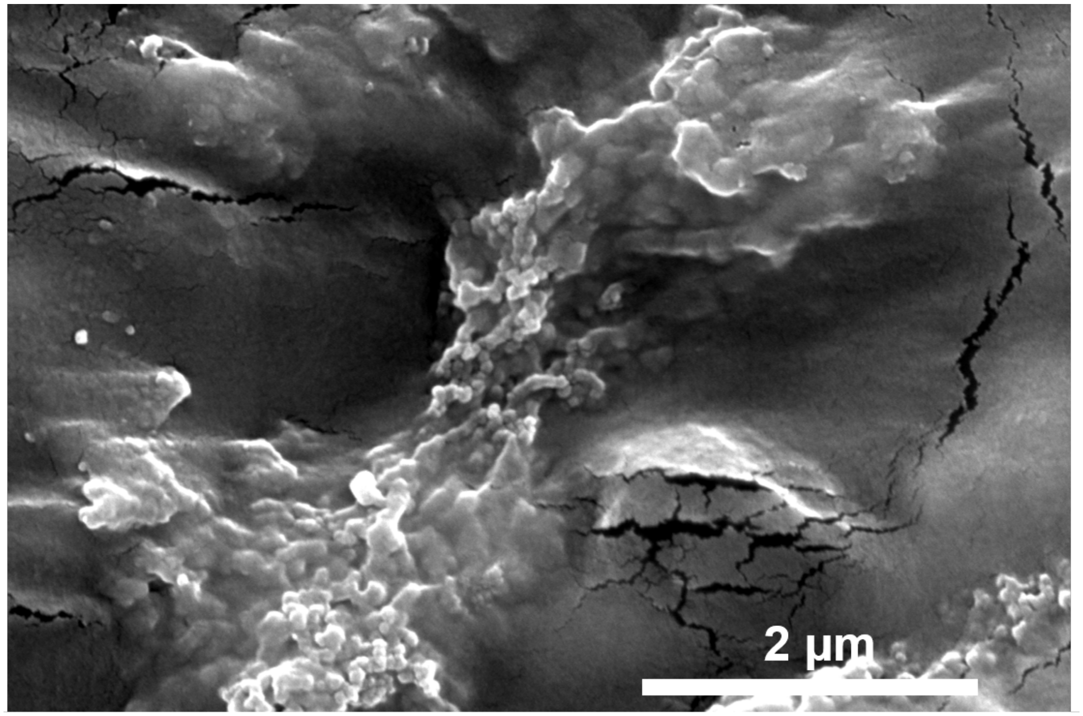

Figure S17. High-magnification SEM image of PVA-MOF-525 composite gel shows the presence of spherical particles, suggesting the formation of MOF-525 in the presence of PVA.

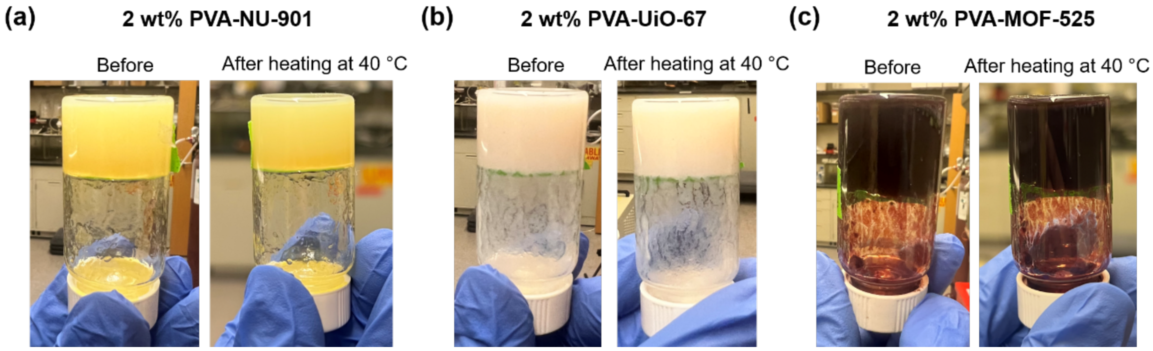

Figure S18. (a) PVA-NU-901, **(b)** PVA-UiO-67, and **(c)** PVA-MOF-525 composite gels stability at room temperature and after heating to 40 °C for 24 h.

Section S3: Composite gel solvent exchange

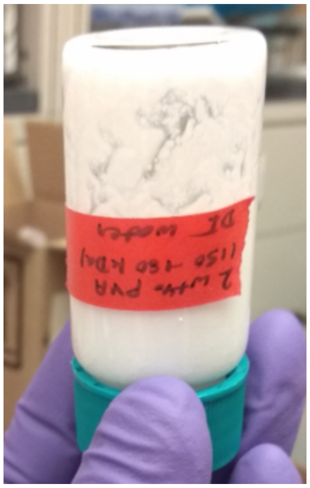

Figure S19. Gelation behavior of reagents used for the 2 wt% PVA-UiO-66 composite, when synthesized in DI water instead of DMSO. The inversion test indicates no gelation in water.

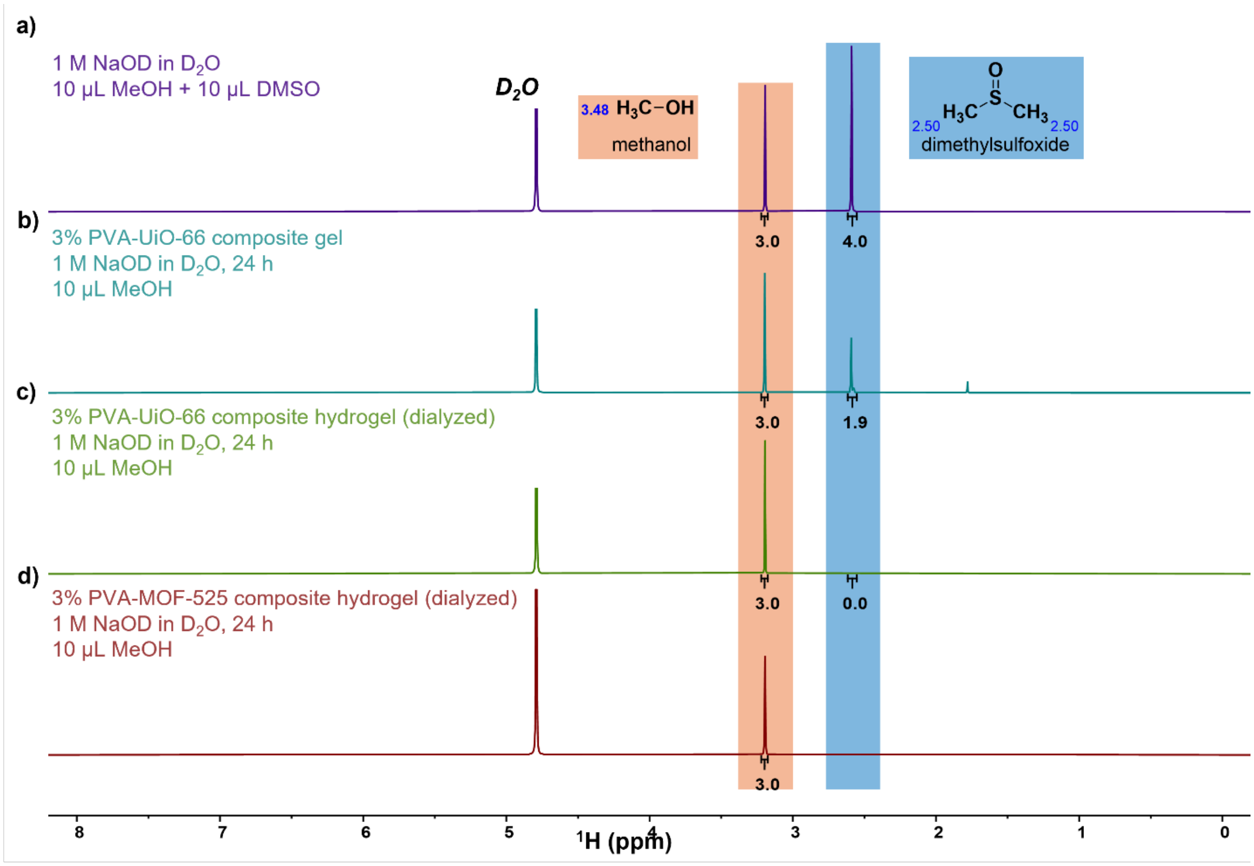

Figure S20. Quantification of dimethylsulfoxide (DMSO) removal during composite gel solvent switch. ^{1}H NMR (400 MHz) of a) 1M sodium deuteroxide (NaOD) in deuterium oxide (D_2O), spiked with 10 μL of methanol (MeOH) and 10 μL of DMSO, b) 3% PVA-UiO-66 composite gel, c) 3% PVA-UiO-66 composite hydrogel (after dialysis), and d) 3% PVA-MOF-525 composite hydrogel (after dialysis). All gel samples were stirred in 1 M NaOD (10 mg gel/mL solvent) for 24 h to solubilize them and were then spiked with 10 μL of MeOH as a reference to determine the concentration of DMSO in each gel. Spectra were referenced to D_2O (4.79 ppm).

Table S1. Quantification of DMSO in polymer-MOF composite hydrogels.

Sample:		3% PVA-UiO-66 Composite Gel	3% PVA-UiO-66 composite hydrogel (dialyzed)	3% PVA-MOF-525 composite hydrogel (dialyzed)
MOF Gel Weight	mg	26	18	30
MeOH amount	mmol	0.25		
MeOH Peak Integration		3.00	3.00	3.00
DMSO Peak Integration		1.90	0.00	0.00
DMSO:MeOH Ratio		0.63	N/A	N/A
DMSO amount	mg	5.07	0	0
	wt%	20%	0%	0%

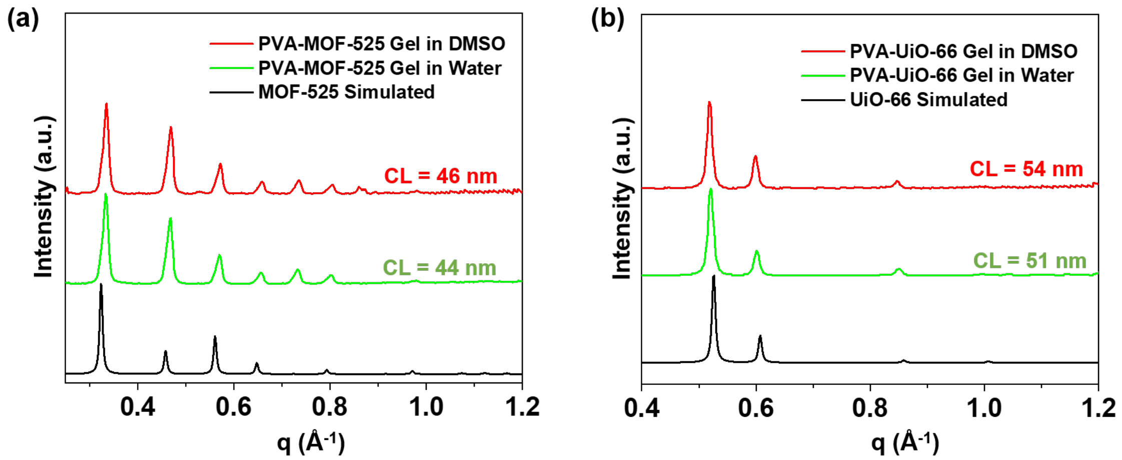

Figure S21: GIXD patterns of 3 wt% **(a)** PVA-MOF-525 and **(b)** PVA-UiO-66 composite gels in DMSO (red) and hydrogels in water (green), showing the MOFs to exhibit similar coherence lengths (CLs) before and after the 48 h solvent exchange (dialysis) process.

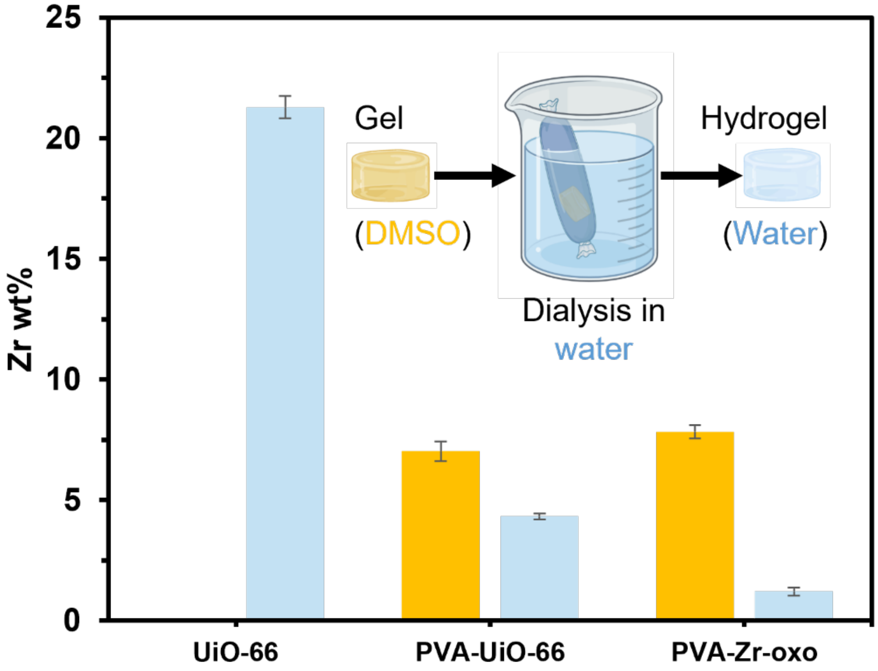

Figure S22. Zr content before and after solvent exchange of PVA-UiO-66 and PVA-Zr-oxo gels from DMSO (yellow) into water (blue) by dialysis, as determined by TGA in air after drying samples at room temperature for 24 h. Zr content decreased significantly in the PVA-Zr-oxo gels during dialysis, but not in the PVA-UiO-66 composite hydrogels. UiO-66 prepared in the absence of polymer contained 21.3 wt% Zr following dialysis. Zr content is reported as an average of 3 samples, with error bars representing the standard deviation across sample sets.

Table S2. ZrO_2 and Zr wt% of PVA-UiO-66 composite gels, PVA-Zr-oxo gels, and UiO-66 powder before and after solvent exchange

		Before Dialysis		After Dialysis	
		**% ZrO_2**	**% Zr**	**% ZrO_2**	**%Zr**
**PVA-UiO-66 composite gel**	1	9.1	6.7	5.8	4.3
	2	10.4	7.7	5.7	4.2
	3	9.9	7.3	6.0	4.4
	**Average**	**9.8**	**7.3**	**5.8**	**4.3**
	**St. Dev.**	**0.7**	**0.5**	**0.2**	**0.1**
**PVA-Zr-oxo gel**	1	10.5	7.8	1.7	1.2
	2	11.0	8.2	1.9	1.4
	3	10.3	7.6	1.4	1.0
	**Average**	**10.6**	**7.8**	**1.6**	**1.2**
	**St. Dev.**	**0.4**	**0.3**	**0.2**	**0.2**
**UiO-66**	1	-	-	28.3	20.9
	2	-	-	29.5	21.8
	3	-	-	28.6	21.1
	**Average**	**-**	**-**	**28.8**	**21.3**
	**St. Dev.**	**-**	**-**	**0.6**	**0.5**

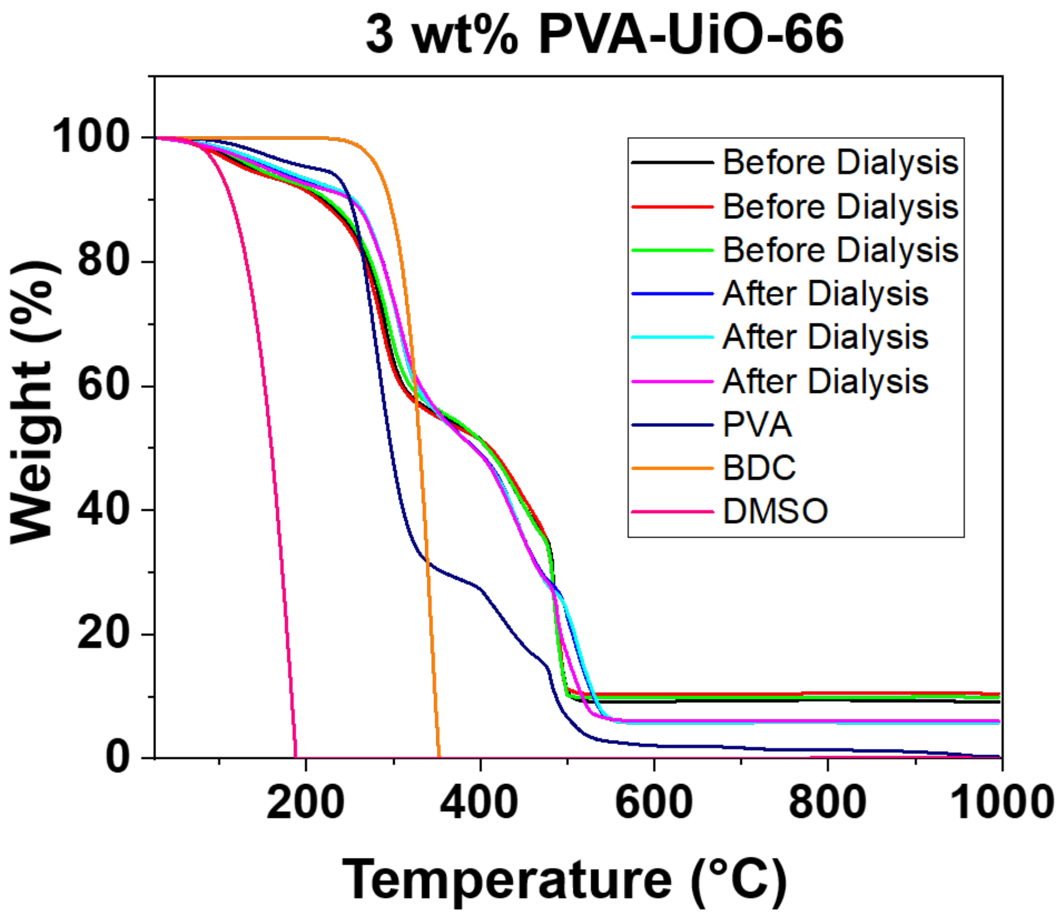

Figure S23. TGA profiles of 3 wt% PVA-UiO-66 composite gels before (black, red, green) and after dialysis (blue, cyan, magenta), BDC (orange), PVA (navy blue), BDC linker (orange) and DMSO (pink). PVA, BDC, and DMSO completely decompose at 1000 °C, suggesting that all the organic components in the PVA-UiO-66 composite gels have been completely removed at 1000 °C and the remaining wt% can be attributed to the formation of ZrO_2. After dialyzing the 3 wt% PVA-UiO-66 composite gels, the ZrO_2 wt% slightly decreases from 9.8 wt% to 5.8 wt%. All the samples were run under air.

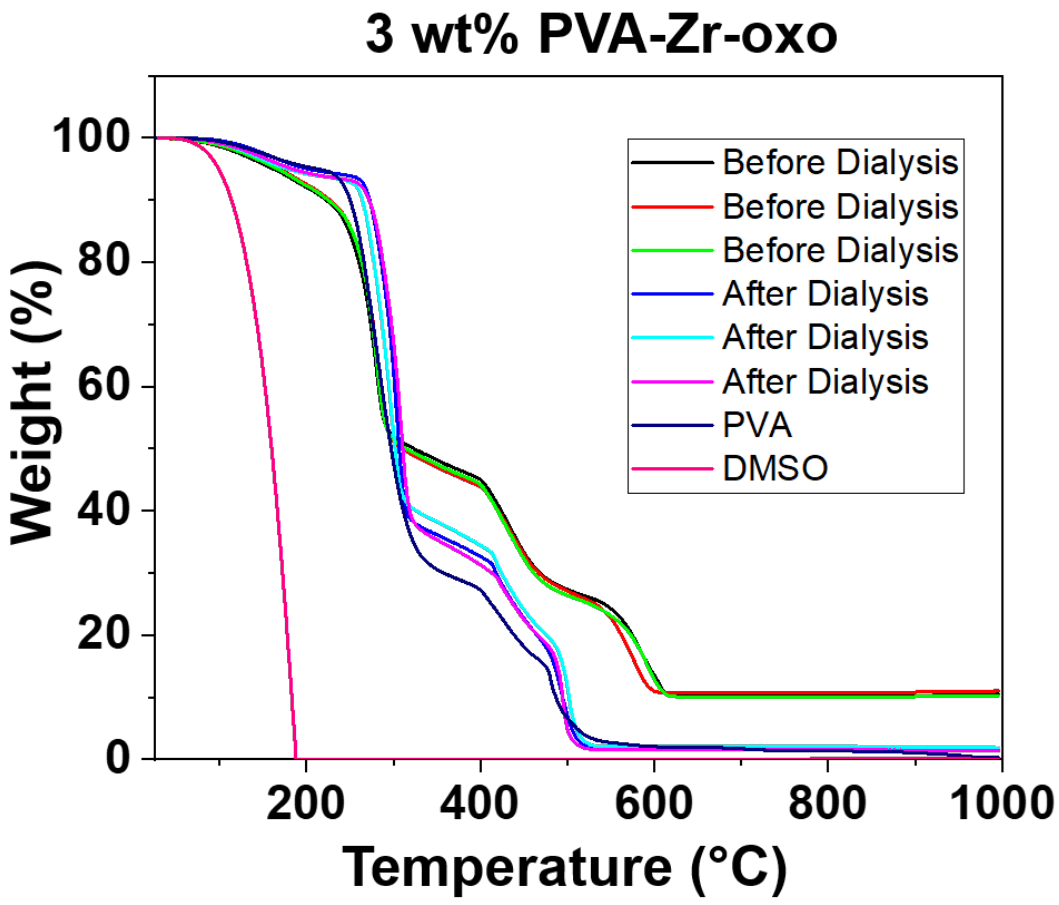

Figure S24. TGA profiles of 3 wt% PVA-Zr-oxo gels before (black, red, green) and after dialysis (blue, cyan, magenta), PVA (navy blue), and DMSO (pink). PVA and DMSO completely decompose at 1000 °C, suggesting that all the organic components in the PVA-UiO-66 composite gels have been completely removed at 1000 °C and the remaining wt% can be attributed to the formation of ZrO_2. After dialyzing the 3 wt% PVA-Zr-oxo gels, the ZrO_2 wt% significantly decreases from 10.6 wt% to 1.6 wt%. All the samples were run under air.

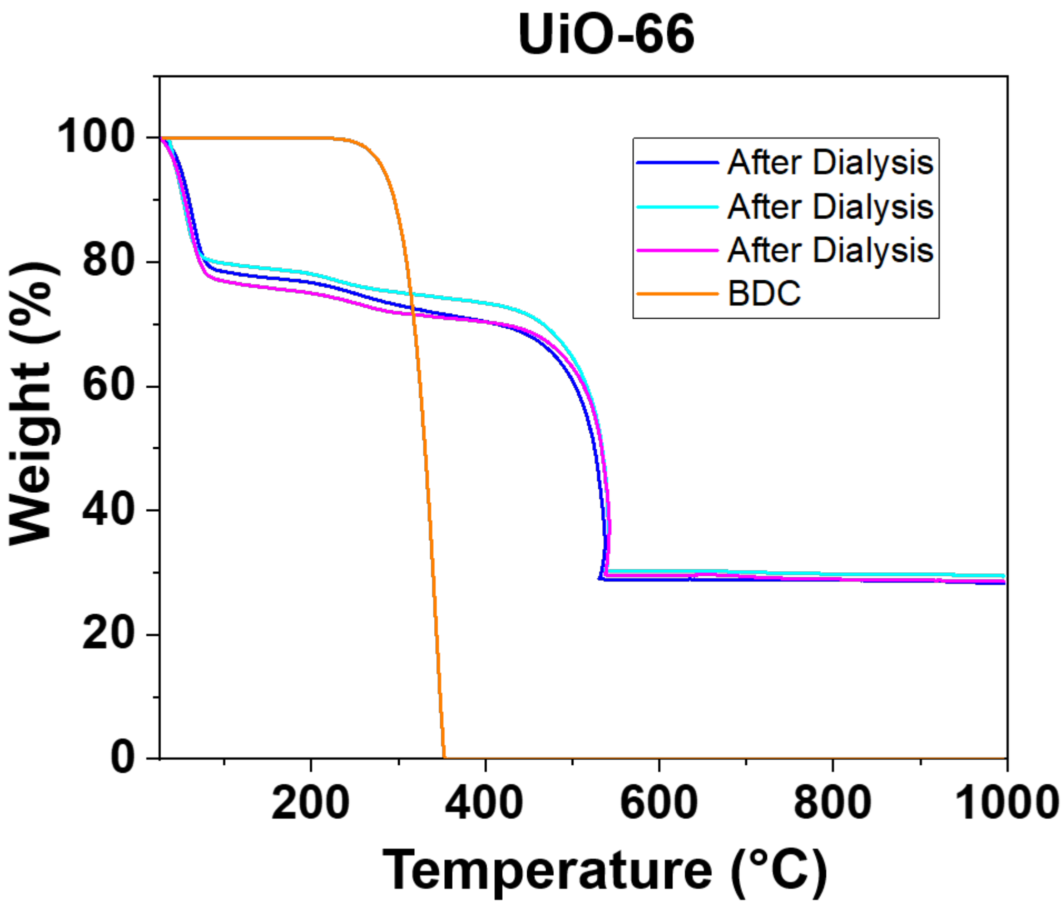

Figure S25. TGA profiles of UiO-66 powder synthesized in DMSO (blue, cyan, magenta) and BDC linker (orange). BDC completely decomposes at 1000 °C, suggesting that the remaining wt% 1000 °C can be attributed to the formation of ZrO_2. All the samples were run under air.

Section S4: MB sorption into and release from PVA-MOF composite gels

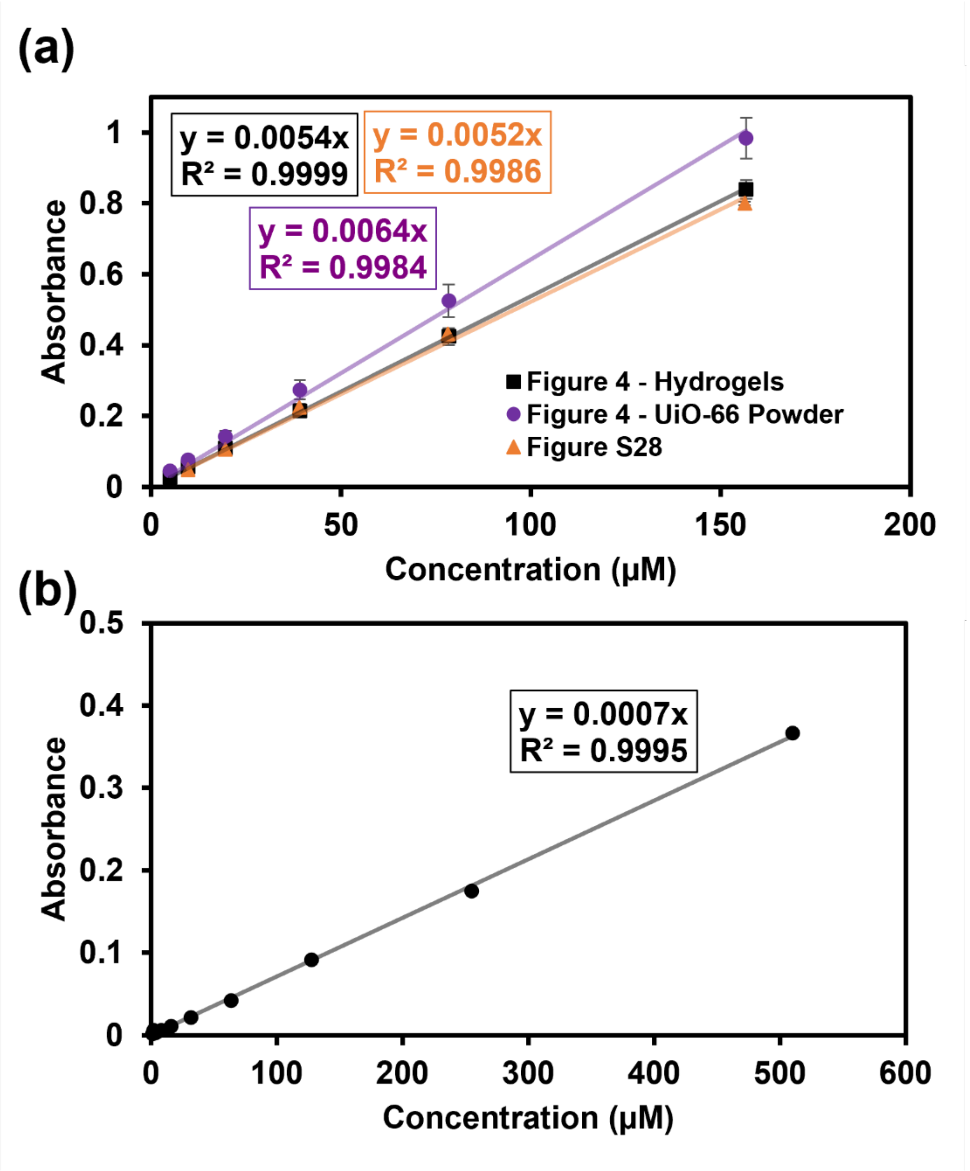

Figure S26. Calibration curves for **(a)** MB and **(b)** Ang 1-7 encapsulation and release experiments into the composite gel carriers and their respective controls (Zr-cross-linked gels and MOF alone). **(a)** MB calibration curves for MB sorption into 3 wt% PVA-UiO-66 composite hydrogels and 3 wt% PVA-Zr-oxo hydrogels (**Figure 4**, **Figures S26-27, and Table S3)**, developed from serial dilution of the respective stock solutions used in the encapsulation experiments. A separate calibration curve was developed for every MB stock solution (all prepared at 0.05 mg/mL) prepared for the experiments: one for the hydrogels (black, squares) one for UiO-66 powder (purple, circles), and one for **Figure S28**, (orange, triangles). Absorbance values were measured at 660 nm, the absorbance maximum for MB. **(b)** Calibration curve for Ang 1-7, developed from serial dilution of the stock solution used in the encapsulation experiments. Absorbance values were measured

at 277 nm, the measured absorbance maximum of Ang 1-7. All stock solutions were prepared in DI water. The absorbance conversion constant ($\varepsilon_s b$) is shown as the slope of the linear interpolations for each dataset, and is listed in the boxes with corresponding colors to each dataset.

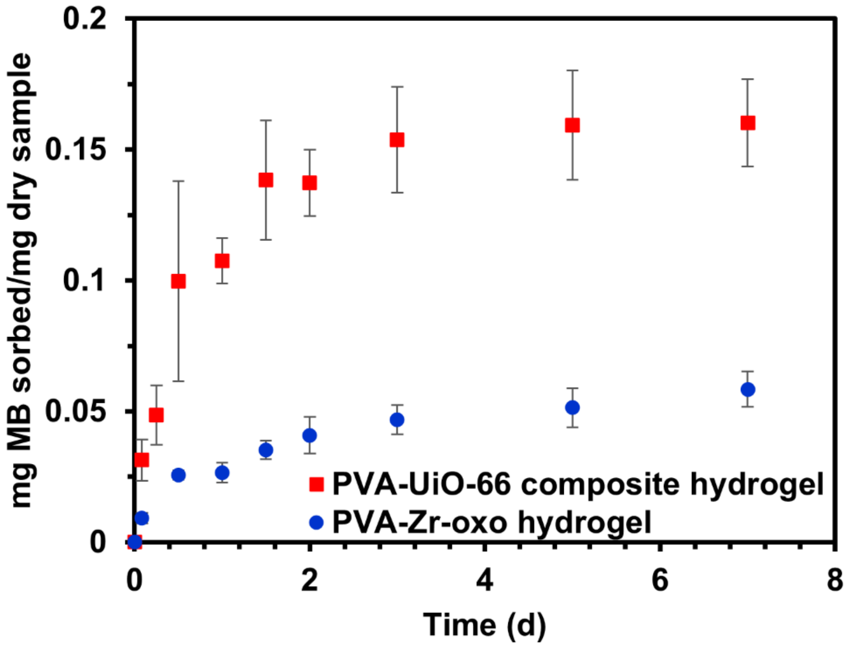

Figure S27. MB sorption profiles of PVA-UiO-66 composite hydrogels (red, squares) and PVA-Zr-oxo hydrogels (blue, circles) over 7 d, normalized by the dry weight of the carrier, showing MB sorption to plateau after 7 d. Points represent the means of three independently synthesized samples, and error bars represent the standard deviation of that mean.

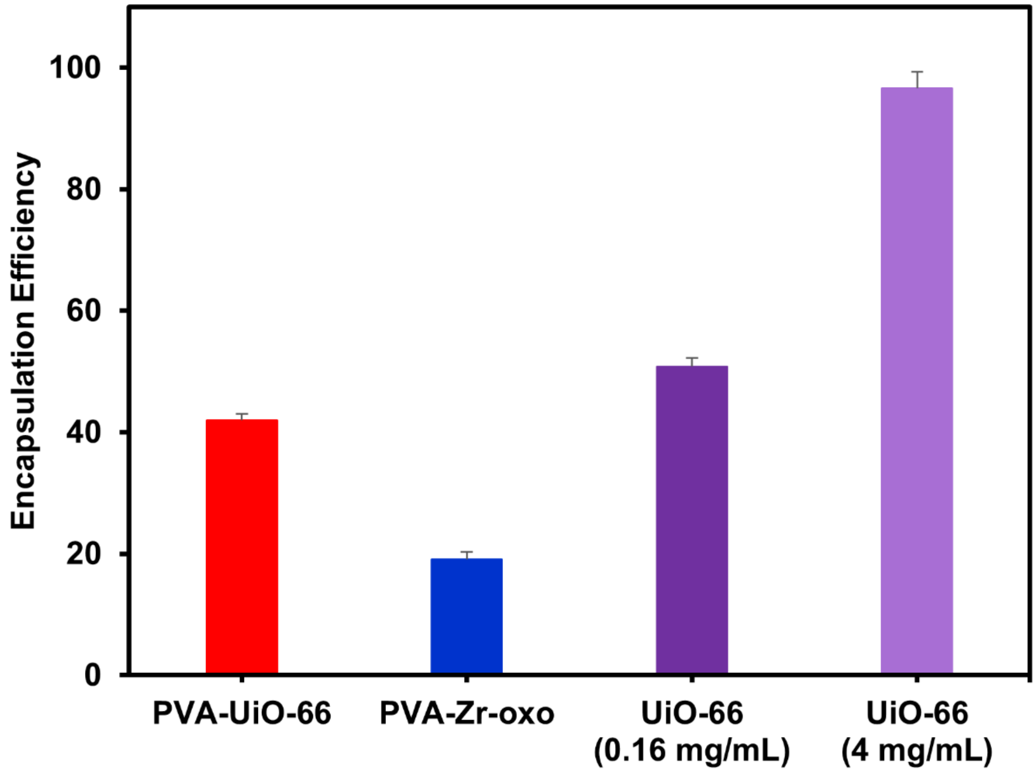

Figure S28. Encapsulation efficiency, or % of MB in solution sorbed, of tested carriers after 7 d. Plot depicts PVA-UiO-66 composite hydrogels (red), PVA-Zr-oxo hydrogels (blue), and UiO-66 (purple). Two separate experiments with UiO-66 are depicted, where the concentration of UiO-66 added into the solution was normalized by total weight (4 mg sample/mL) and by Zr weight (0.16 mg sample/mL), relative to the PVA-UiO-66 composite hydrogels. While the PVA-UiO-66 composite hydrogel, PVA-Zr-oxo hydrogel, and UiO-66 normalized by Zr weight (0.16 mg sample/mL) all reached sorptive capacity after 7 d, as they all sorbed less than 100% of the MB in solution; however, the UiO-66 samples normalized by total weight (4 mg sample/mL) sorbed ~100% of the MB in the solution, suggesting that it was not at sorptive capacity. Therefore, to ensure all analyzed samples were at sorptive capacity, the Zr-weight normalized UiO-66 samples (0.16 mg sample/mL) were used to compare to the gel samples. Plotted bars represent the average of three independently synthesized samples incubated with MB for 7 d. Error bars represent the standard deviation of the average.

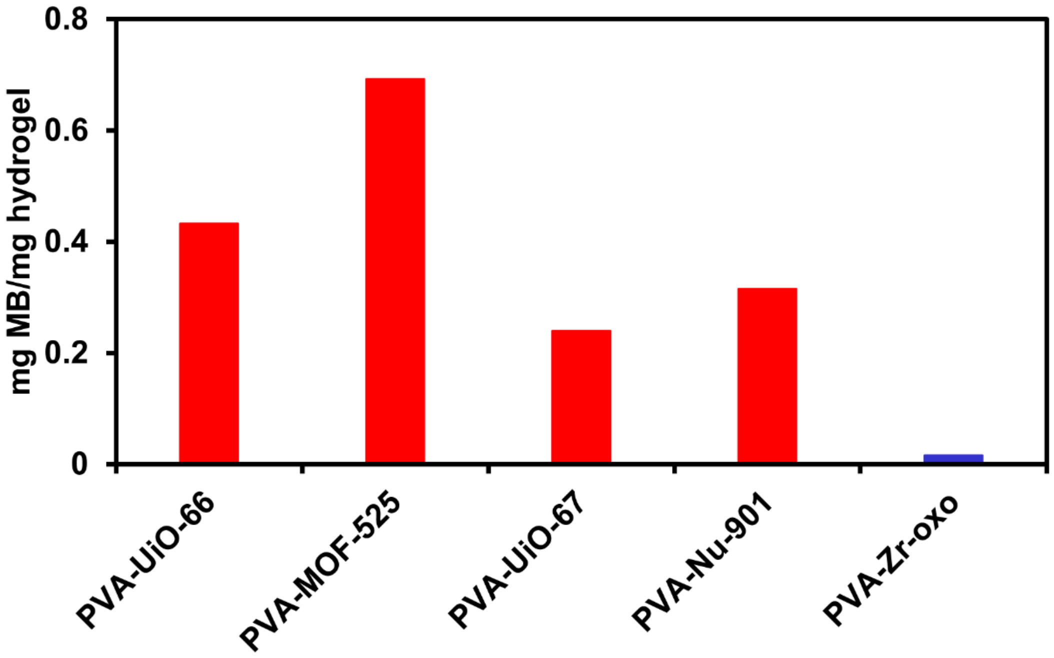

Figure S29. MB sorptive capacity of various PVA-MOF composite hydrogels. Amount of MB sorbed into each PVA-MOF hydrogel shown in Figure 3 (PVA-UiO-66, PVA-MOF-525, PVA-UiO-67, and PVA-NU-901) (red) and PVA-Zr-oxo (blue) hydrogels after 7 d, normalized by the weight of each hydrated gels added to the MB solution. All synthesized PVA-MOF composite hydrogels exhibited higher MB sorptive capacity than the PVA-Zr-oxo control hydrogels. Each gel was synthesized with 2 wt% PVA, and lightly agitated in solution of MB over 7 d.

Table S3. MB sorption into PVA-UiO-66 composite hydrogels, PVA-Zr-oxo hydrogels, and UiO-66 powder.

	**mg MB 1-7/mg carrier**			**mg MB 1-7/mg dry carrier**			**mg MB/mg Zr**		
		**Avg**	**StDev**		**Avg**	**StDev**		**Avg**	**StDev**
**PVA-UiO-66 composite gel**	0.005	0.006	0.001	0.141	0.160	0.017	3.267	3.708	0.385
	0.007			0.172			3.981		
	0.006			0.167			3.875		
**PVA-Zr-oxo gel**	0.002	0.003	0.000	0.052	0.058	0.007	4.324	4.838	0.561
	0.003			0.057			4.752		
	0.003			0.066			5.436		
**UiO-66**	0.157	0.160	0.006	0.157	0.160	0.006	0.731	0.744	0.027
	0.156			0.156			0.727		
	0.167			0.167			0.776		

Table S4. Reported values and conditions for UiO-66 MB sorptive capacities in literature.

Sorptive Capacity (mg MB/g sorbent)	pH	Starting MB concentration (mg/L	Sorbent	Reported by:
64.5	-	50	UiO-66	ref. 45
~544	10	130	UiO-66	ref 46
160 +/- 17	-	50	3% PVA-UiO-66 composite hydrogels	this study
160 +/- 6	-	50	UiO-66	this study

Section S5: Ang 1-7 sorption into and release from PVA-MOF-525 composite gels

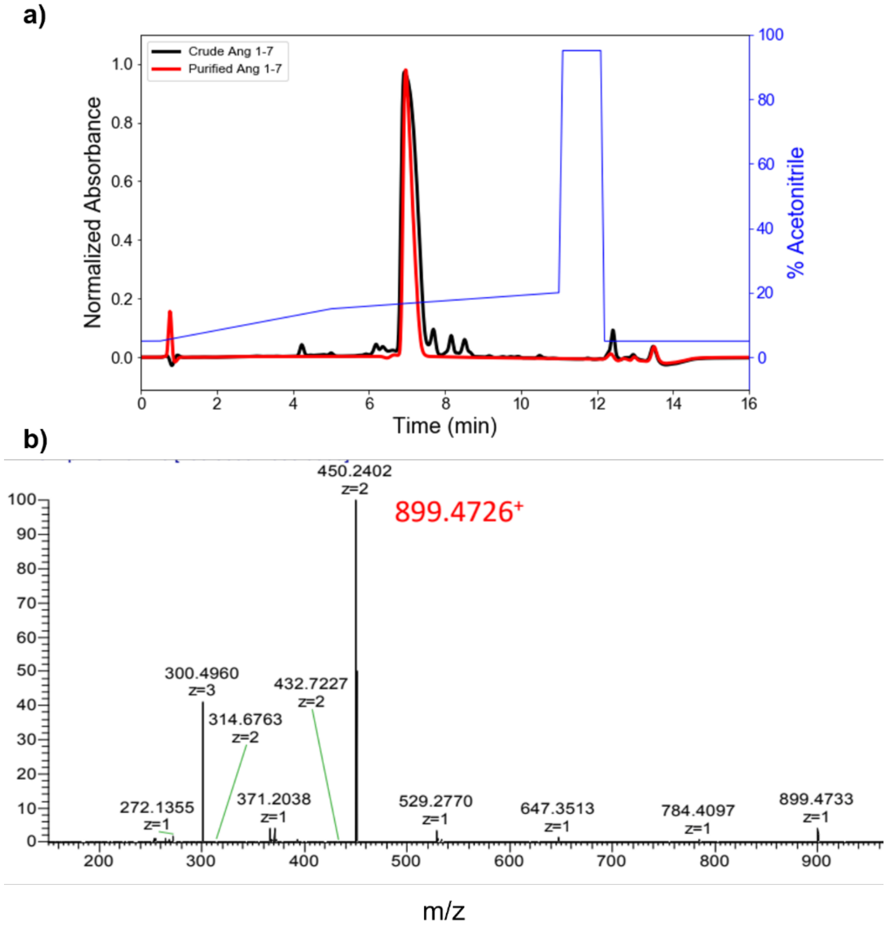

Figure S30: Characterization of Ang 1-7 peptide. **(a)** Analytical reverse phase high performance liquid chromatography (RP-HPLC) of Ang 1-7 before (black) and after purification (red) using preparative scale RP-HPLC. Analytical RP-HPLC was performed on a Waters E2695 Alliance Separations Module, using a 4.5 mm x 50 mm XBridge C18 3.5 µm chromatographic separation column, where the acetonitrile gradient is plotted in blue. Preparative scale RP-HPLC was performed on a Waters Empower system, using a 30 mm x 150 mm XBridge Prep C18 5 µm optimum bed density chromatographic separation columns. UV absorbance was measured at 214 nm. **(b)** Electrospray ionization (ESI) spectrometry of Ang 1-7 after purification. ESI was measured a Thermo Orbitrap Exploris 480 mass spectrometer by the UVA Biomolecular Analysis Core.

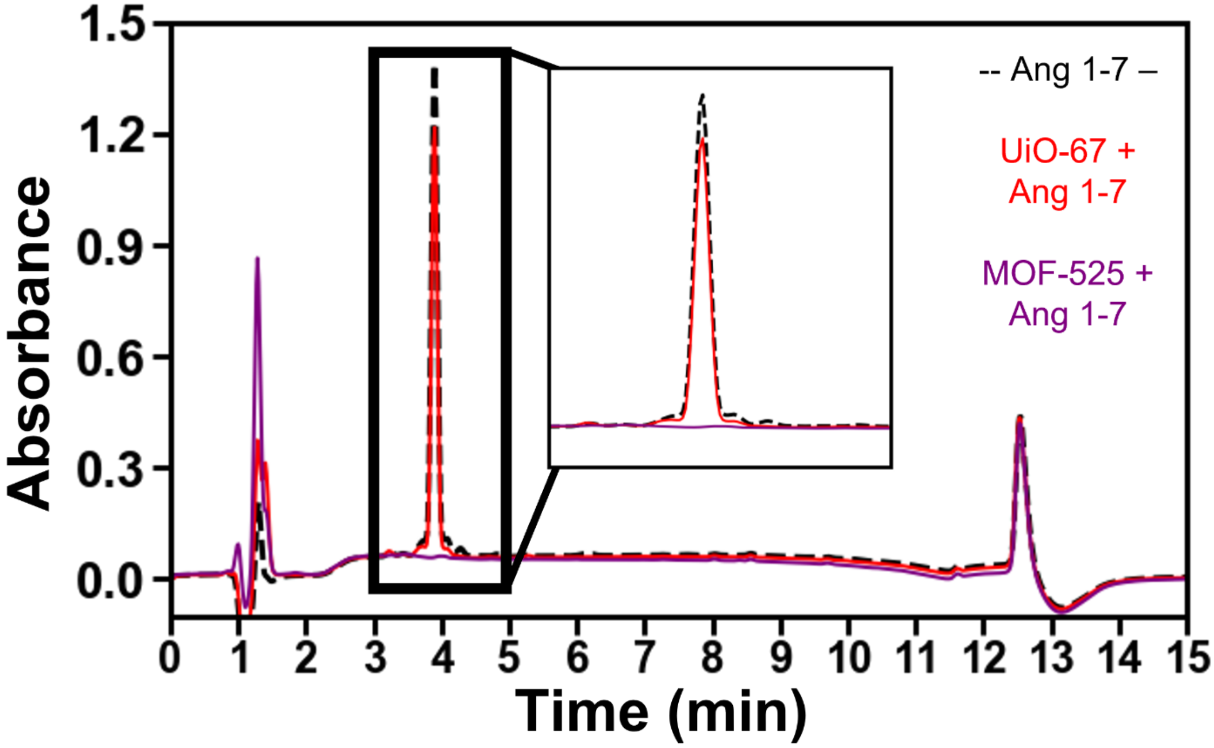

Figure S31: Reverse phase high performance liquid chromatography depicting Ang 1-7 (black) encapsulation into UiO-67 (red) and MOF-525 (purple). Little-to-no decrease in the Ang 1-7 peak (~3.75 m) was detected after incubation with UiO-67, suggesting little-to-no encapsulation of Ang 1-7 in UiO-67. In contrast, the complete disappearance of the Ang 1-7 peak in the sample incubated with MOF-525 suggests encapsulation of Ang 1-7. Therefore, we used MOF-525 based carriers for the Ang 1-7 encapsulation and release studies. Analytical RP-HPLC was performed on a Waters E2695 Alliance Separations Module, using a 4.5 mm x 50 mm XBridge C18 3.5 µm chromatographic separation column. Mobile phase composed of ultrapure water and acetonitrile + 0.1% trifluoroacetic acid, run at a gradient of 15 to 20% ACN from 2 to 12 min. UV absorbance was measured at 214 nm.

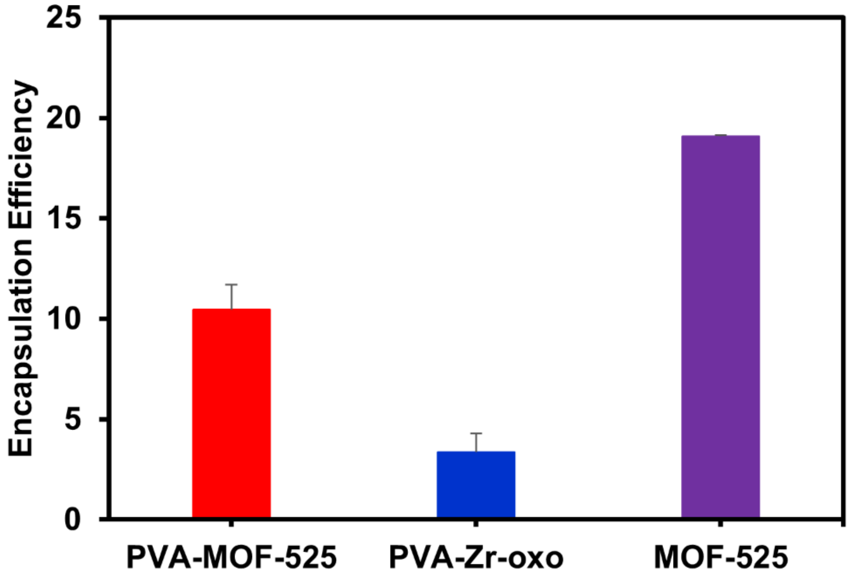

Figure S32: Encapsulation efficiency, or % of Ang 1-7 in solution sorbed, of PVA-MOF 525 composite hydrogels (red), PVA-Zr-oxo hydrogels (blue) and MOF-525 powder (purple). All samples were determined to be at sorptive capacity, as their encapsulation efficiencies were lower than 100%. Plotted bars represent the average of 3 independently synthesized samples incubated with Ang 1-7 for 7 d. Error bars represent the standard deviation of the average.

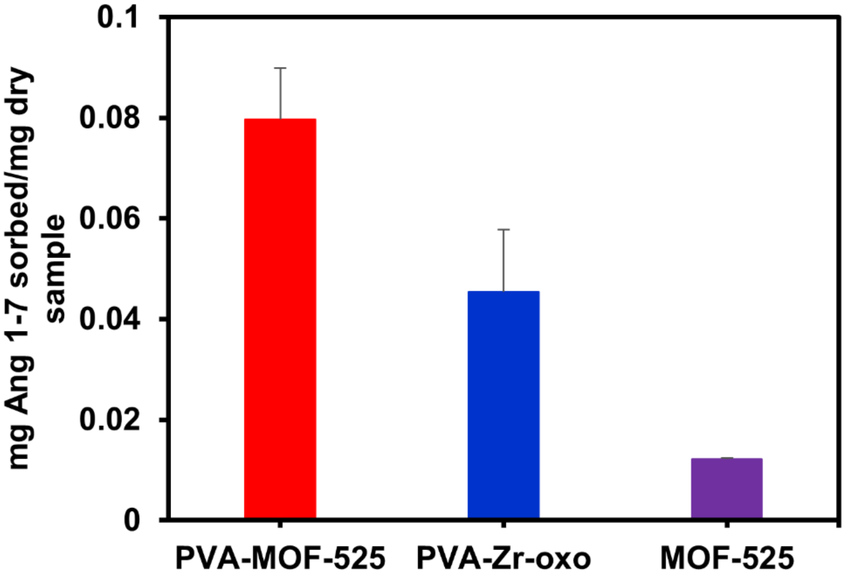

Figure S33. Sorptive capacity of PVA-MOF-525 composite hydrogels (red), PVA-Zr-oxo hydrogels (blue), and MOF-525 powder (purple) for the therapeutic peptide Ang 1-7. **(a)** Amount of Ang 1-7 sorbed into each sample after 7 d, normalized relative to the dry mass of each sample, showing more Ang 1-7 to sorb into the PVA-MOF-525 composite hydrogels than into the PVA-Zr-oxo hydrogels and MOF-525 on a per dry mass basis. Despite normalizing to dry weight, the hydrogels were added to Ang 1-7 solution in a swollen state, and the MOF-525 powder was added in a dry state.

Table S5. Ang 1-7 sorption into PVA-MOF-525 composite hydrogels, PVA-Zr-oxo hydrogels, and MOF-525 powder

	**mg Ang 1-7/mg carrier**			**mg Ang 1-7/mg dry carrier**		
**PVA-MOF-525 composite gel**		**Avg**	**StDev**		**Avg**	**StDev**
	5.7	6.70	0.9	68.2	79.7	10.2
	7.0			83.3		
	7.3			87.6		
**PVA-Zr-oxo**		Avg	StDev		Avg	StDev
	1.7	2.2	0.6	36.2	45.3	12.5
	1.9			40.3		
	2.8			59.5		
**MOF-525**		Avg	StDev		Avg	StDev
	12.0	12.2	0.2	12.0	12.2	0.2
	12.3			12.3		
	12.2			12.2		

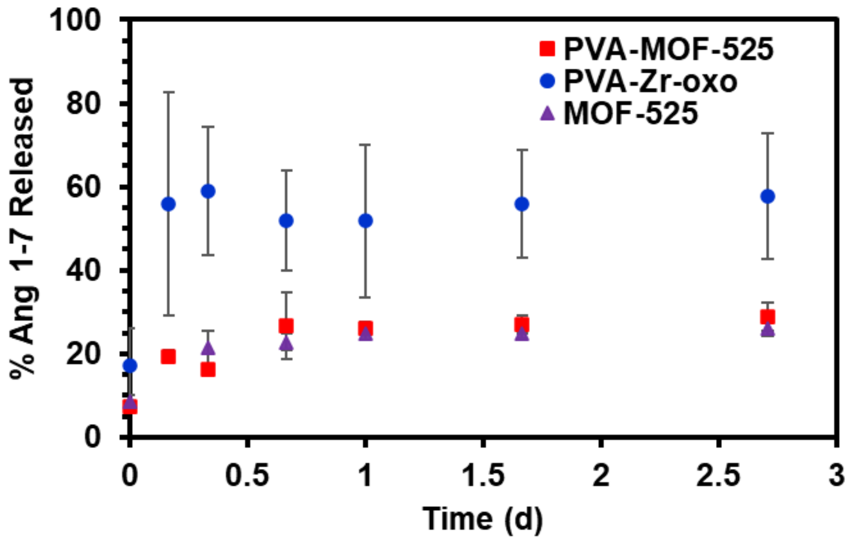

Figure S34. % of Ang 1-7 sorbed into PVA-MOF-525 composite hydrogels (red, square), PVA-Zr-oxo hydrogels (blue, circle), and MOF-525 powder (purple, triangle) that was released into ultrapure water.

Table S6. % of Ang 1-7 sorbed into PVA-MOF-525 composite hydrogels, PVA-Zr-oxo hydrogels, and MOF-525 powder released into ultrapure water.

Time (d)	PVA-MOF-525		PVA-Zr-oxo		MOF-525	
	Average	StDev	Average	StDev	Average	StDev
0.0	7.4	0.7	17.1	8.9	8.7	1.4
0.2	19.3	1.5	56.0	26.7		
0.3	16.3	0.8	58.9	15.3	21.5	4.1
0.7	26.8	8.0	51.9	12.1	22.9	2.0
1.0	26.1	1.5	51.8	18.2	25.0	1.6
1.7	27.0	2.1	55.8	12.8	24.9	1.1
2.7	28.8	3.3	57.7	14.9	26.1	2.0

References

(1) Wang, T. C.; Vermeulen, N. A.; Kim, I. S.; Martinson, A. B. F.; Stoddart, J. F.; Hupp, J. T.; Farha, O. K. Scalable Synthesis and Post-Modification of a Mesoporous Metal-Organic Framework Called NU-1000. *Nat. Protoc.* **2016**, *11* (1), 149–162. https://doi.org/10.1038/nprot.2016.001.

(2) Destefano, M. R.; Islamoglu, T.; Garibay, S. J.; Hupp, J. T.; Farha, O. K. Room-Temperature Synthesis of UiO-66 and Thermal Modulation of Densities of Defect Sites. *Chem. Mater.* **2017**, *29*, 1357-1361. https://doi.org/10.1021/acs.chemmater.6b05115.

(3) Huelsenbeck, L.; Luo, H.; Verma, P.; Dane, J.; Ho, R.; Beyer, E.; Hall, H.; Geise, G. M.; Giri, G. Generalized Approach for Rapid Aqueous MOF Synthesis by Controlling Solution pH. *Cryst. Growth Des.* **2020**, *20* (10), 6787–6795. https://doi.org/10.1021/acs.cgd.0c00895.

(4) Islamoglu, T.; Otake, K.-I.; Li, P.; Buru, C. T.; Peters A. W.; Akpinar, I.; Garibay, S. J.; Farha, O. K. Revisiting the Structural Homogeneity of NU-1000, a Zr-Based Metal–Organic Framework. *CrystEngComm* **2018**, *20* (39). 5913-5918. https://doi.org/10.1039/c8ce00455b.

(5) Thompson, A. P.; Aktulga, H. M.; Berger, R.; Bolintineanu, D. S.; Brown, W. M.; Crozier, P. S.; in 't Veld, P. J.; Kohlmeyer, A.; Moore, S. G.; Nguyen, T. D.; Shan, R.; Stevens, M. J.; Tranchida, J.; Trott, C.; Plimpton, S. J. LAMMPS - a Flexible Simulation Tool for Particle-Based Materials Modeling at the Atomic, Meso, and Continuum Scales. *Comput. Phys. Commun.* **2022**, *271*, 108171. https://doi.org/10.1016/j.cpc.2021.108171.

(6) Boyd, P. G.; Moosavi, S. M.; Witman, M.; Smit, B. Force-Field Prediction of Materials Properties in Metal-Organic Frameworks. *J. Phys. Chem. Lett.* **2017**, *8* (2), 357–363. https://doi.org/10.1021/acs.jpclett.6b02532.

(7) Rappé, A. K.; Casewit, C. J.; Colwell, K. S.; Goddard, W. A.; Skiff, W. M. UFF, a Full Periodic Table Force Field for Molecular Mechanics and Molecular Dynamics Simulations. *J. Am. Chem. Soc.* **1992**, *114* (25), 10024–10035. https://doi.org/10.1021/ja00051a040.

(8) Darve, E.; Rodríguez-Gómez, D.; Pohorille, A. Adaptive Biasing Force Method for Scalar and Vector Free Energy Calculations. *J. Chem. Phys.* **2008**, *128* (14), 144120. https://doi.org/10.1063/1.2829861.

(9) Sidky, H.; Colón, Y. J.; Helfferich, J.; Sikora, B. J.; Bezik, C.; Chu, W.; Giberti, F.; Guo, A. Z.; Jiang, X.; Lequieu, J.; Li, J.; Moller, J.; Quevillon, M. J.; Rahimi, M.; Ramezani-Dakhel, H.; Rathee, V. S.; Reid, D. R.; Sevgen, E.; Thapar, V.; Webb, M. A.; Whitmer, J. K.; De Pablo, J. J. SSAGES: Software Suite for Advanced General Ensemble Simulations. *J. Chem. Phys.* **2018**, *148* (4), 044104. https://doi.org/10.1063/1.5008853.

(10) Vanden-Eijnden, E.; Venturoli, M. Revisiting the Finite Temperature String Method

for the Calculation of Reaction Tubes and Free Energies. *J. Chem. Phys.* **2009**, *130* (19), 194103. https://doi.org/10.1063/1.3130083.

(11) Sarkisov, L.; Bueno-Perez, R.; Sutharson, M.; Fairen-Jimenez, D. Materials Informatics with PoreBlazer v4.0 and the CSD MOF Database. *Chem. Mater.* **2020**, *32* (23), 9849–9867. https://doi.org/10.1021/acs.chemmater.0c03575.

(12) Patterson, A. L. The Scherrer Formula for X-Ray Particle Size Determination. *Phys. Rev.* **1939**, *56* (10), 978–982. https://doi.org/10.1103/PhysRev.56.978.

(13) Athar, M.; Rzepka, P.; Thoeny, D.; Ranocchiari, M.; Anton Van Bokhoven, J. Thermal Degradation of Defective High-Surface-Area UiO-66 in Different Gaseous Environments. *RSC Adv.* **2021**, *11* (61), 38849–38855. https://doi.org/10.1039/d1ra05411b.

(14) Dighe, A. V.; Huelsenbeck, L.; Bhawnani, R. R.; Verma, P.; Stone, K. H.; Singh, M. R.; Giri, G. Autocatalysis and Oriented Attachment Direct the Synthesis of a Metal-Organic Framework. *JACS Au* **2022**, *2* (2), 453–462. https://doi.org/10.1021/jacsau.1c00494.

(15) Pascual-Colino, J.; Artetxe, B.; Beobide, G.; Castillo, O.; Fidalgo-Mayo, M. L.; Isla-López, A.; Luque, A.; Mena-Gutiérrez, S.; Pérez-Yáñez, S. The Chemistry of Zirconium/Carboxylate Clustering Process: Acidic Conditions to Promote Carboxylate-Unsaturated Octahedral Hexamers and Pentanuclear Species. *Inorg. Chem.* **2022**, *61* (12), 4842–4851. https://doi.org/10.1021/acs.inorgchem.1c03466.
